# Targeting the alternative sigma factor RpoN to combat virulence in *Pseudomonas aeruginosa*

**DOI:** 10.1038/s41598-017-12667-y

**Published:** 2017-10-03

**Authors:** Megan G. Lloyd, Benjamin R. Lundgren, Clayton W. Hall, Luke B.-P. Gagnon, Thien-Fah Mah, Jennifer F. Moffat, Christopher T. Nomura

**Affiliations:** 1 Department of Microbiology and Immunology, SUNY Upstate Medical University, Syracuse, NY USA; 2 Department of Chemistry, SUNY College of Environmental Science and Forestry, Syracuse, NY USA; 3 Center for Applied Microbiology, SUNY College of Environmental Science and Forestry, Syracuse, NY USA; 4 Department of Biochemistry, Microbiology, and Immunology, University of Ottawa, Ottawa, Ontario, Canada

## Abstract

*Pseudomonas aeruginosa* is a Gram-negative, opportunistic pathogen that infects immunocompromised and cystic fibrosis patients. Treatment is difficult due to antibiotic resistance, and new antimicrobials are needed to treat infections. The alternative sigma factor 54 (σ^54^, RpoN), regulates many virulence-associated genes. Thus, we evaluated inhibition of virulence in *P. aeruginosa* by a designed peptide (RpoN molecular roadblock, RpoN*) which binds specifically to RpoN consensus promoters. We expected that RpoN* binding to its consensus promoter sites would repress gene expression and thus virulence by blocking RpoN and/or other transcription factors. RpoN* reduced transcription of approximately 700 genes as determined by microarray analysis, including genes related to virulence. RpoN* expression significantly reduced motility, protease secretion, pyocyanin and pyoverdine production, rhamnolipid production, and biofilm formation. Given the effectiveness of RpoN* *in vitro*, we explored its effects in a *Caenorhabditis elegans–P. aeruginosa* infection model. Expression of RpoN* protected *C. elegans* in a paralytic killing assay, whereas worms succumbed to paralysis and death in its absence. In a slow killing assay, which mimics establishment and proliferation of an infection, *C. elegans* survival was prolonged when RpoN* was expressed. Thus, blocking RpoN consensus promoter sites is an effective strategy for abrogation of *P. aeruginosa* virulence.

## Introduction

*Pseudomonas aeruginosa* is an opportunistic pathogen that causes disease in soft tissue, burns, and in immunocompromised individuals, with wound infection rates from 17% to 59% in hospitals worldwide^1–3^. Cystic fibrosis patients are particularly susceptible to *P. aeruginosa* infections, with almost 80% of adults infected^4^. *P. aeruginosa* is naturally resistant to many antibiotics, including certain penicillins and cephalosporins^5^, easily acquires resistance through mutations or acquisition of genes and has been identified on a recently released WHO “Global Priority List” as a critical pathogen and top priority for research and development of new antibiotics^6^. According to the CDC, increased antibiotic resistance in *P. aeruginosa* limits effective treatments in hospital-acquired infections, highlighting the need for novel antimicrobials. Preventing the expression or activity of virulence factors has emerged as a promising approach to identify and develop novel agents that would impair the ability of *P. aeruginosa* to cause disease^7–11^.

*P. aeruginosa* controls gene expression through 24 sigma factors, each with a defined regulon^12^. Multiple sigma factors are involved in controlling global regulators which, in turn, regulate expression of virulence factors^13^. The alternative sigma factor, σ^54^ or RpoN, was initially discovered as part of the nitrogen utilization pathway, but is now associated with regulation of many virulence factors^12^, including motility^14,15^, quorum sensing^16,17^, mucoidy^18^, and biofilms^19^. In fact, motility, mucoidy, and quorum sensing are under dual regulation with RpoN and another transcriptional regulator^18,20,21^. RpoN is also associated with *P. aeruginosa* virulence in nematodes and mice^22^. Furthermore, some genes necessary for host infection have been identified as part of the RpoN regulon, including *vfr, kinB*, and *rhlR* ^23,24^. While not all virulence factors are universally required to infect potential hosts, some are common for infections in both humans and *Caenorhabditis elegans* ^23^.

RpoN has a highly conserved Region III that recognizes a unique −24/−12 promoter with the consensus sequence TGGC-N_9_-GC^12,25,26^. Notably, the −24 element, which is centered on the conserved ‘GG’ dinucleotides, is specifically recognized by a motif known as the ‘RpoN box’ which is located in the C-terminal portion of RpoN^27^. A study by Doucleff, *et al*. demonstrated that a peptide comprised of the C-terminal 60-amino acids of the *Aquifex aeolicus* RpoN protein specifically binds the -24 element with high affinity (K_d_~109 nM)^27^. This interaction is considered a main driving force for promoter recognition by RpoN. Additionally, unlike other sigma factors, RpoN can bind its DNA sequence without RNA polymerase (RNAP), although binding is 10-fold less efficient than the RpoN:RNAP complex^28^.

Here, we describe an engineered peptide, RpoN molecular roadblock or RpoN*, that antagonizes gene transcription by binding to RpoN specific promoters to reduce expression of RpoN-related *P. aeruginosa* virulence factors. RpoN* is identical to the 60 aa C-terminal DNA binding domain of *A. aeolicus*, except for a methionine to initiate translation^27^. The molecular roadblock was first evaluated for its effects on gene transcription in *P. aeruginosa*. The phenotype of RpoN* expression was then evaluated *in vitro* and *in vivo* using a *P. aeruginosa–C. elegans* infection model. We report that the roadblock reduced transcription of many virulence-related genes, which was verified in relevant *in vitro* assays. Furthermore, the roadblock improved survival of *C. elegans* exposed to *P. aeruginosa*. These results demonstrate that effective binding of an engineered peptide to RpoN consensus promoters is a novel and effective method for reducing *P. aeruginosa* virulence.

## Results

### Engineering the molecular roadblock RpoN*

**Figure 1:**
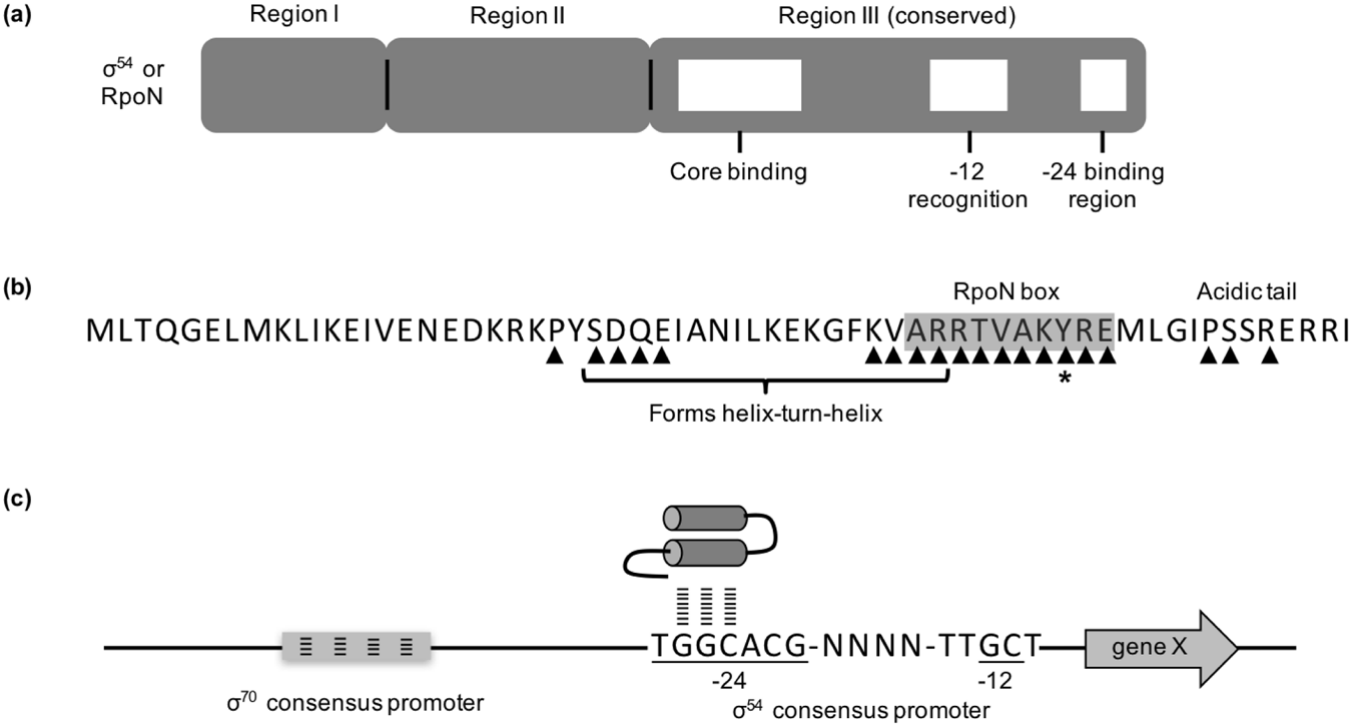
Schematic of σ^54^ (RpoN) and the molecular roadblock, RpoN*. (**a**) σ^54^ (RpoN) is composed of three regions. Region III is highly conserved, and is necessary for recognizing and binding −24/−12 promoter elements. (**b**) Amino acid sequence of RpoN*. Peptide sequence includes AA376-400 of RpoN Region III from *A. aeolicus* and includes amino acids (▴) that specifically bind to the −24 promoter DNA. Point mutation (Y48A, asterisk) attenuates binding and transcriptional activity of RpoN*. (**c**) Schematic of the interaction between RpoN* and the −24 element of the σ^54^ consensus promoter.

We expected that a peptide consisting of only the C-terminal domain of RpoN would be sufficient to antagonize genome-wide transcription from RpoN consensus promoters. Therefore, a gene encoding a peptide resembling the last 60 amino acids of RpoN from the thermophile *A. aeolicus* was synthesized and codon-optimized for expression in *E. coli* (Fig. 1). This gene, rpoN*, was cloned into a broad-host range plasmid under an inducible *trc*-promoter for controlled expression (see Supplementary Table S1 for a list of plasmids and oligonucleotides). The sequence of RpoN* has previously been shown to specifically bind −24 RpoN consensus promoter sites^27^. When used with transcriptomic profiling and predictive modeling, RpoN* was expected to help identify sites of RpoN regulation, including points of regulation not previously detected, such as genes with multiple promoters and/or under negative control by RpoN.

### RpoN* affects gene transcription

**Figure 2:**
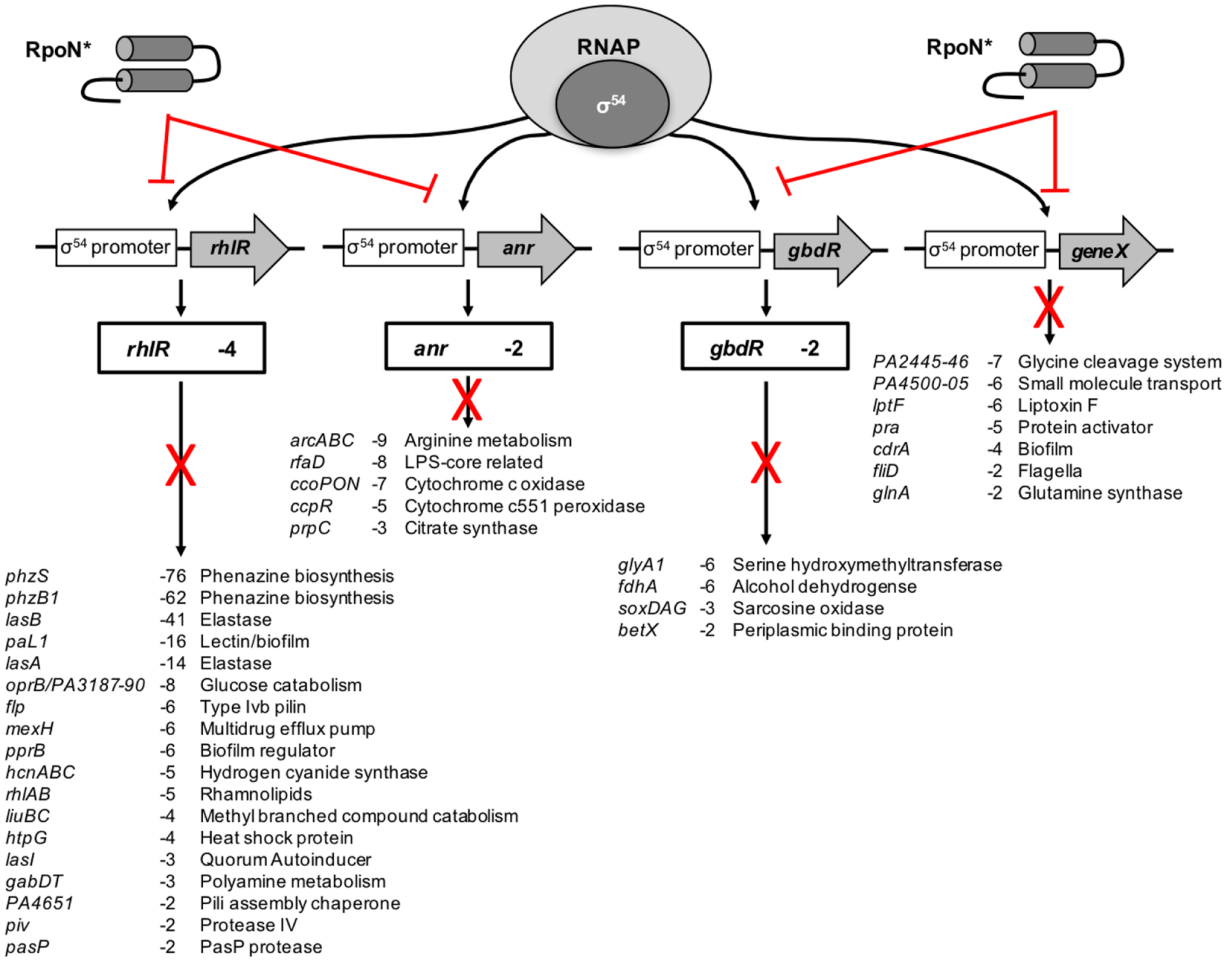
Summary of genes affected by expression of RpoN* in *P. aeruginosa* PAO1. Expression of RpoN* interferes with the ability of RNA polymerase and native RpoN to bind to sig54 promoters altering the transcriptional profile of *P. aeruginosa* PAO1. Summaries of areas of gene expression that are specifically altered by RpoN* are shown and include the genes encoding the transcriptional regulators RhlR, Anr, GbdR, and their respective regulons. These results are consistent with RpoN binding sites identified by ChIP-seq.^24^. Expression of RpoN* was shown to alter expression of a number of other genes including hypothetical (~250) and ribosomal (~70) genes. A full list of these genes is available in Supplementary Table S2.

To determine the effects RpoN* has on gene transcription, microarray analysis was performed. *P. aeruginosa* PAO1 and an isogenic *ΔrpoN* strain were transformed with an empty vector or vector expressing RpoN*. The bacteria were grown to mid-exponential phase in rich media with IPTG to induce RpoN* expression, and then RNA was purified and analyzed using commercially available genome arrays (Agilent). Overall, approximately 700 genes were differentially transcribed at least 2-fold in the presence of RpoN* (Supplementary Table S2; full data available at GEO accession: GSE35632). Most were downregulated (>400), whereas the majority of upregulated genes involved lipopolysaccharide (LPS) and ribosomal biosynthesis. A selection of genes affected by the roadblock are displayed in Fig. 2. These genes encode proteins involved in metabolism, virulence, stress/survival, or cell signaling. Some genes affected by the RpoN* molecular roadblock have been shown to directly bind RpoN^24^. A few of these genes encode transcriptional regulators with their own regulons, including those of the *las* and *rhl* quorum signaling systems^29–31^, and the transcription factors *anr* ^32^ and *gbdR* ^33^. Many genes in each of these signaling regulons were also affected by the roadblock, as would be expected (Fig. 2). The expression of the native *rpoN* gene was also reduced by approximately one-third, which is expected as *rpoN* has a consensus promoter for its gene product^24^.

Deletion of native RpoN altered transcription of over 1,800 genes by 1.5-fold or more, with most of these genes downregulated (>950) (Supplementary Table S3, first column) (GEO Accession: GSE35632). Prior studies suggest that multiple sigma factors can bind promoter sites and control transcription of a single gene in *P. aeruginosa* ^12,16,24^. For instance, deletion of RpoN did not affect the levels of *rhlR* transcription because RpoS, a σ^70^-like factor, can also bind a −35/−10 consensus promoter at the same gene and regulate transcription^24^. A large overlap was observed between gene transcription downregulated by RpoN* in PAO1 and loss of RpoN in the *ΔrpoN* mutant (Supplementary Fig. S1). However, some genes were exclusively regulated by the roadblock, suggesting RpoN* reduced transcription of genes under dual regulation. When the microarray analysis of the *ΔrpoN* mutant harboring the empty vector was compared to *ΔrpoN* expressing RpoN*, more than 300 genes were differentially transcribed 1.5-fold or more (Supplementary Table S3, second column). Again, RpoN* altered transcription of global regulators, including *rhlR*, *vfr* and *anr*, subsequently affecting genes in their respective regulons demonstrating the specificity of this peptide to bind to RpoN promoter sequences (Supplementary Table S3, second column). Additional comparisons and overlap of genes identified for the different conditions in the microarray is shown in Supplementary Fig. S1.

**Figure 3:**
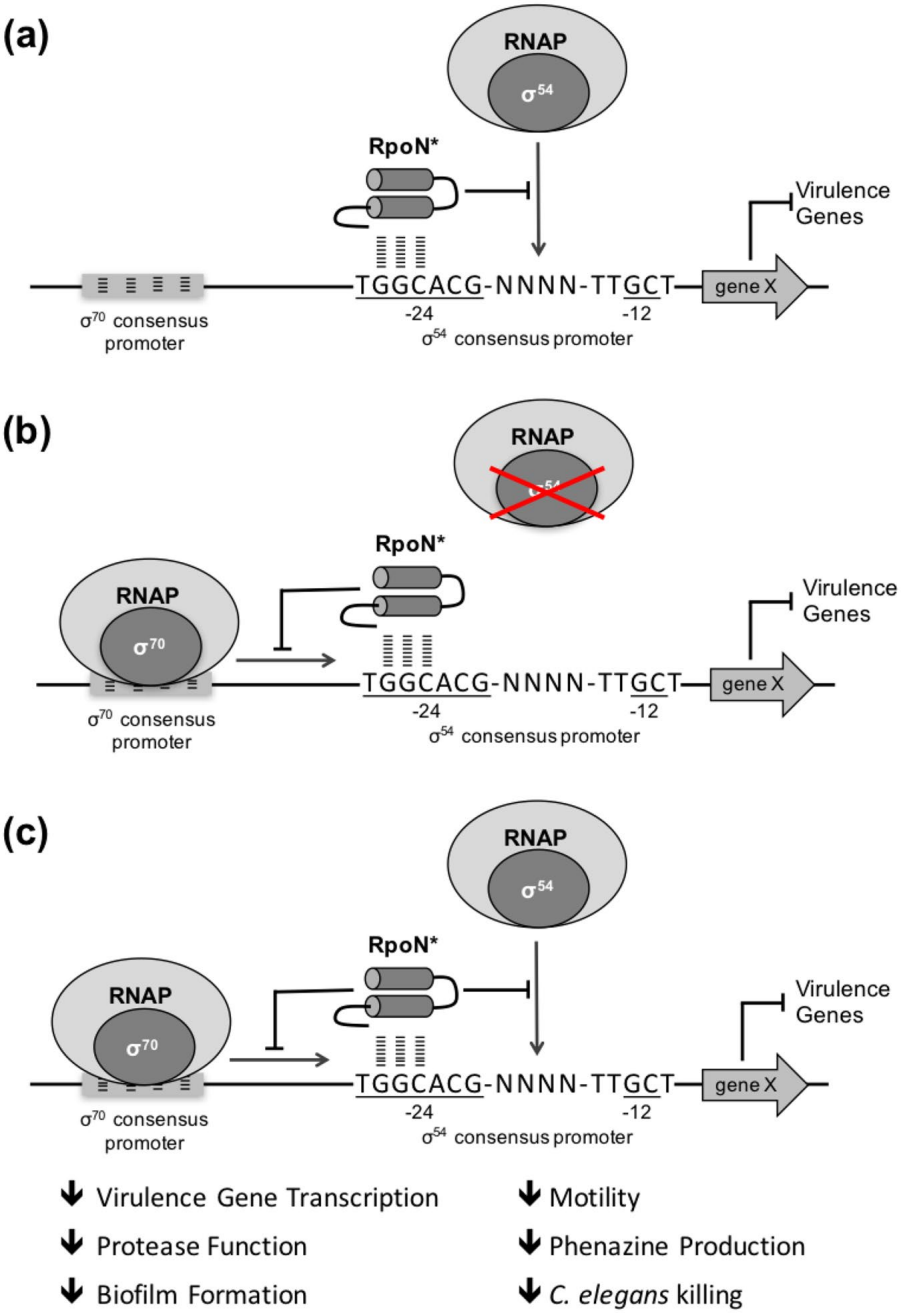
Model of predicted RpoN* mechanism of action. (**a**) RpoN*, a cis-acting peptide, binds -24 promoter sites, blocking gene transcription by RpoN. (**b**) Additional sigma factors can compensate for absence of native RpoN, resulting in transcription of associated genes. In the absence of native RpoN, the RpoN* blocks transcription by other sigma factors with a promoter binding site at the same gene. (**c**) RpoN* blocks transcription by obstructing RpoN:RNAP binding, as well as halting transcription of other sigma factors binding at the same gene.

Based on the microarray analysis, we propose a model for the RpoN* mechanism of action (Fig. 3). The expected mechanism of action of the cis-acting molecular roadblock is that it binds at the −24 site of RpoN consensus promoters, blocking RpoN from binding, thus reducing gene expression at these sites, as well as blocking downstream signaling effects (Fig. 3a). However, in the absence of native RpoN, gene transcription was also reduced. Thus, we also propose that the molecular roadblock can bind −24 consensus promoter sites, blocking transcription by other sigma factors with −35/−10 consensus promoter sites at the same gene (Fig. 3b). Furthermore, unlike full-length RpoN, RpoN* cannot interact with native RNAP, so we expect expression of RpoN* to minimally impact the transcriptional machinery of *P. aeruginosa*.

### RpoN* reduces virulence phenotypes

**Figure 4:**
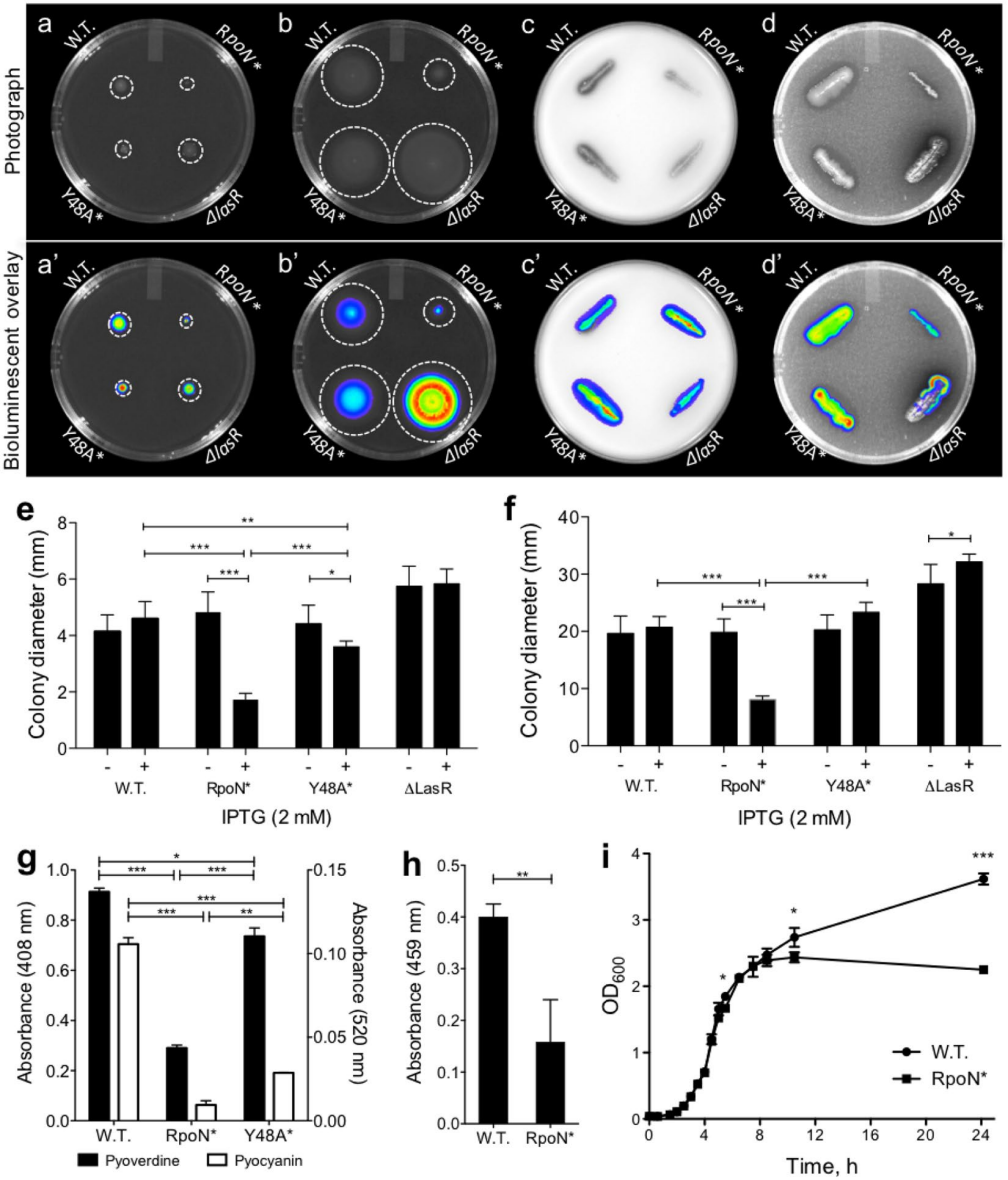
RpoN* expression reduced virulence phenotypes. (**a**–**d**) Photograph (top) plus bioluminescent overlay (bottom) for phenotype assays: (**a**,**a’**) twitching, or pili, motility conducted on semi-hard agar; (**b**,**b’**) swimming, or flagellar, motility assays conducted on soft agar; (**c**,**c’**) protease assays conducted on milk agar; (**d**,**d’**) elastase assays conducted on LB agar with elastin. Strains used: *P. aeruginosa* PA19660 **(﻿a-f**) and PAO1 (**g-i**) wild-type (empty vector), RpoN*, Y48A* point mutant, and *P. aeruginosa* PAO1 Δ*lasR*. All assays were conducted at 37 °C for 24–48 h, with 30 mg/L gentamicin and 2 mM IPTG. Colony diameter of *P. aeruginosa* strains in twitching (**e**) and swimming (**f**) motility assays, with (+) and without (−) 2 mM IPTG. Pyocyanin and pyoverdine (**g**) production assays conducted in LB or King’s B broth, respectively, with 30 mg/L gentamicin and 1 mM IPTG. Elastase (**h**) production assay in peptone-tryptic soy broth with 1 mM IPTG. Growth kinetics (**i**) with RpoN* expression induced with 1 mM IPTG at 0.5 OD_600_. Statistics used were Student’s t-test (***p ≤ 0.0001; **p ≤ 0.01; *p ≤ 0.05). Bars indicate mean colony diameter of replicates; error bars represent one standard deviation of the mean. n = 4 to 7 replicates per assay.

Genes associated with motility, proteases, pyoverdine biosynthesis, phenazine production, and rhamnolipid production were found to have lower transcript levels in the presence of RpoN*. We validated these findings by evaluating *P. aeruginosa* harboring the empty vector, RpoN*, or the variant Y48A in RpoN*, with an expectation that if RpoN* reduced expression of specific genes, decreases in the associated phenotypes would be observed. The tyrosine residue of the RpoN Box is crucial for DNA binding^34^, therefore it was expected that the Y48A variant would be less effective than RpoN* at antagonizing RpoN-related functions. Two wild type *P. aeruginosa* strains were tested: PAO1 and PA19660, a bioluminescent strain to better visualize the bacteria in the motility and protease assays. The negative control for virulence was *P. aeruginosa ΔlasR*:*gent* ^*R*^ that was not transformed with plasmids. As expected, *P. aeruginosa* harboring the empty vector plasmid was motile and exhibited protease activity (Fig. 4). *P. aeruginosa ΔlasR* significantly increased motility but lacked protease and elastase activity (Fig. 4). Introduction of RpoN* significantly decreased swimming and twitching motility (Students t-test, p ≤ 0.0001) (Fig. 4). Additionally, expression of RpoN* reduced protease activity, and significantly decreased extracellular levels of the siderophore pyoverdine, the phenazine pyocyanin (Students t-test, p ≤ 0.0001), as well as elastase (Students t-test, p ≤ 0.01). RpoN* expression also reduced rhamnolipid production (Supplementary Fig. S2). Finally, induction of RpoN* in a liquid culture modestly reduced growth rate, with significant differences between growth for only a few time points (Student’s t-test, at 5.5 h and 10.5 h p ≤ 0.05, at 24 h p ≤ 0.0001). In comparison, Y48A RpoN* significantly reduced *P. aeruginosa* twitching motility (Student’s t-test, p ≤ 0.05), pyocyanin production (Students t-test, p ≤ 0.001), and pyoverdine production (Students t-test, p ≤ 0.05), but did not affect swimming motility, or protease activity. The intermediate effects of Y48A RpoN* were expected due to its reduced binding affinity for the −24 element of the RpoN promoter. The RpoN* molecular roadblock was also effective at reducing virulence-associated phenotypes in the *P. aeruginosa* Δ*rpoN* mutant (Supplementary Fig. S3, see Supplementary Note for detailed explanation). These results support the findings from our microarray study that demonstrated that RpoN* could effectively reduce transcription of genes involved in the production of various virulence factors in *P. aeruginosa*.

### RpoN* decreases biofilm formation *in vitro*

**Figure 5:**
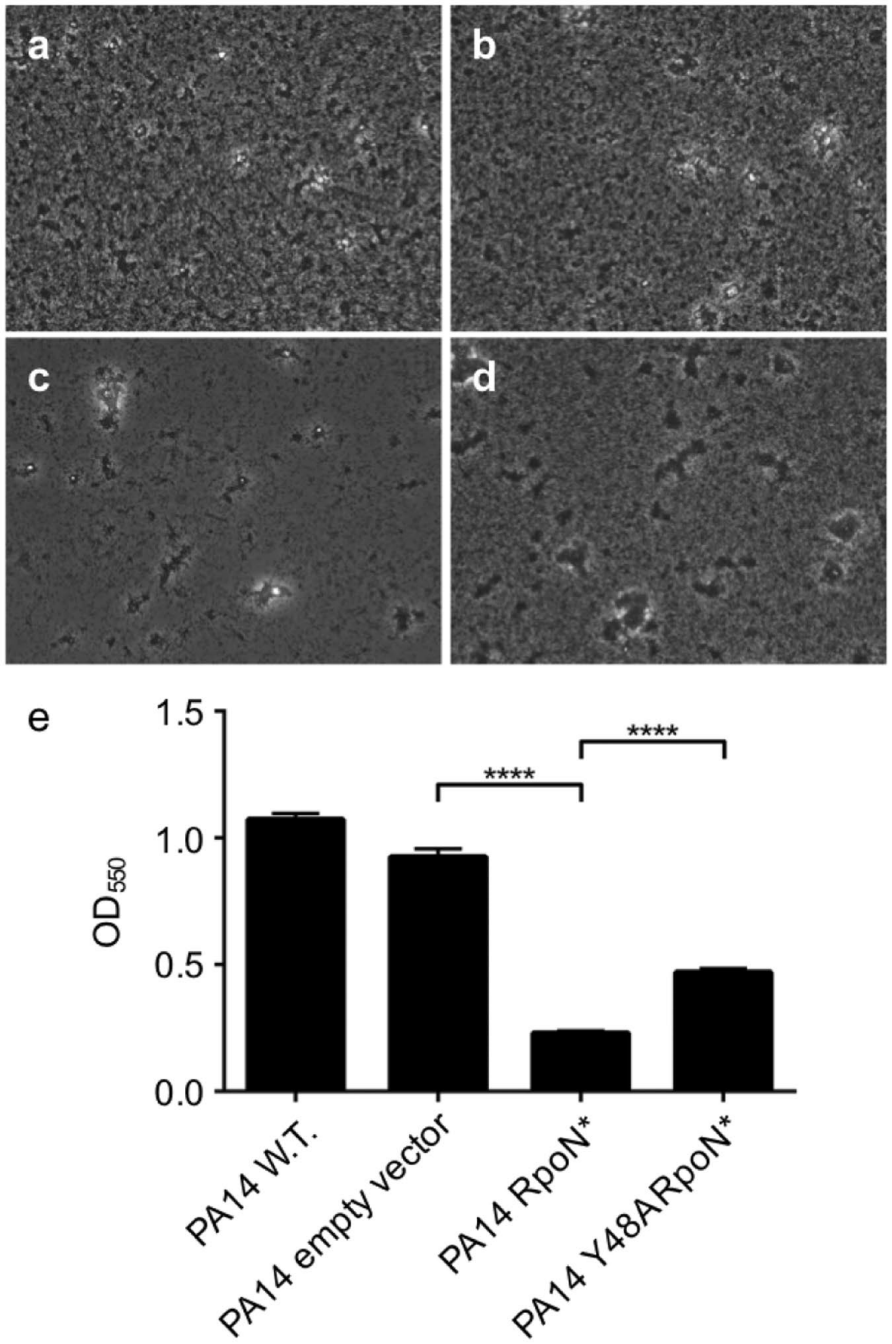
RpoN* decreases biofilm formation *in vitro*. (**a**–**d**) Air-Liquid Interface (ALI) biofilm formation assay conducted in well bottoms of 6-well microtiter plates for 24 h and visualized by phase contrast microscopy at 200x. Strains used: *P. aeruginosa* PA14 wild type (**a**), empty vector (**b**), RpoN* (**c**), and Y48A* point mutant (**d**). Images shown are representative fields of view from two independent experiments with three technical replicates for each strain. (**e**) Biofilms formed in 96-well microtiter plates were stained with crystal violet, solubilized, and quantified at OD_550_ (n = 28). Data presented as mean ± SEM. Student’s *t* test performed (****p ≤ 0.0001).

Biofilm formation is a major obstacle in treating many bacterial infections. Microarray analysis revealed that RpoN* reduced transcription of genes involved in biofilm formation, including *cdrA*, *paL1*, and *pprB*. Additionally, a recent study on sigma-factor regulons in *P. aeruginosa* PA14 identified the biofilm-related *pel* genes as having a RpoN binding site^24^. Thus, we sought to confirm if RpoN* could hinder biofilm formation *in vitro* using both an air-liquid interface (ALI) assay and a microtiter-dish based assay. Wild type *P. aeruginosa* PA14 was used as a positive control. The test conditions were *P. aeruginosa* wild-type (empty vector), RpoN*, or the point mutant Y48A RpoN*. If RpoN* reduced expression of genes involved in biofilm formation, we expected to see diminished formation of biofilms *in vitro*. As expected, wild-type *P. aeruginosa* PA14 formed dense biofilms (Fig. 5a,b,e), with or without the empty vector plasmid. Conversely, *P. aeruginosa* expressing RpoN* diminished biofilm formation in both the ALI (Fig. 5c) and microtiter-dish assays (Fig. 5e; Student’s t-test p ≤ 0.0001). Expression of the Y48A RpoN* also reduced biofilm formation, but to a lesser extent than RpoN* (Fig. 5d,e; Student’s t-test p ≤ 0.0001). These results are consistent with RpoN* acting as an antagonist of biofilm formation in *P. aeruginosa*.

### RpoN* increases *C. elegans* survival in paralytic killing assay

Phenotypic assays and microarray analysis indicated that expression of RpoN* could repress production of virulence factors in *P. aeruginosa*. Thus, the effects of the roadblock were next evaluated with a *P. aeruginosa–C. elegans* infection model. *C. elegans* is an efficient *in vivo* model for evaluating *P. aeruginosa* virulence, as *C. elegans* has innate defenses to protect from bacteria, some of which have homologs in the human innate system^35^. Additionally, *C. elegans* is often used for screening antimicrobials^36^. Thus, this model is a valuable tool to study the effects of RpoN* in *P. aeruginosa* virulence. Each infection model, or killing strategy, involves different virulence factors, with varied types of media used to promote each specific killing strategy^37,38^. This is in accordance with other Gram-negative bacteria, where choice of media is known to affect gene expression^39^.

**Figure 6:**
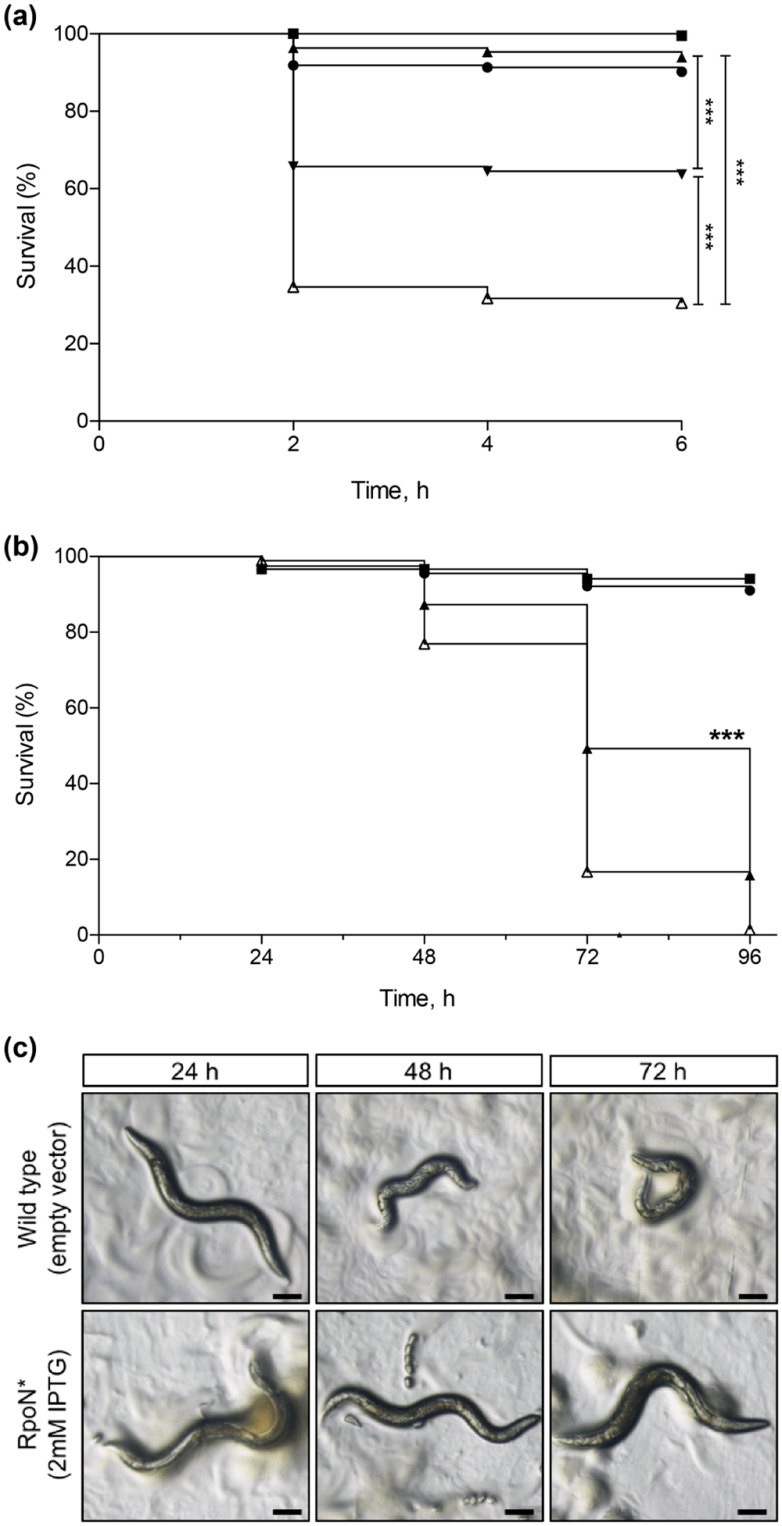
RpoN* increases *C. elegans* survival in *C*. *elegans* – *P*. *aeruginosa* infection assays. (**a**) Brain-heart infusion media with bacto-agar was supplemented with 30 mg/L gentamicin and at least 1 mM IPTG. All paralytic killing assays were conducted at room temperature (22–24 °C), and scored every 2 h. Strains used: *E. coli* (▪, n = 195), *P. aeruginosa* Δ*lasR* (⚫, n = 197), wild-type *P. aeruginosa* PAO1 (Manoil strain) (empty vector, Δ, n = 205), RpoN* (▴, n = 301), and Y48A* point mutant (▾, n = 376). (**b**) Slow killing agar (0.35% bactopeptone, 2% bactoagar) was supplemented with 30 mg/L gentamicin and at least 1 mM IPTG, when necessary. Slow killing assays were conducted at 20 °C and scored every 24 h. Strains used: *E. coli* (▪, n = 90), *P. aeruginosa* Δ*lasR* (⚫, n = 90), wild-type *P. aeruginosa* PA19660 *Xen5* (empty vector, Δ, n = 171), RpoN* (▴, n = 160). (**c**) Appearance of worms fed on *P. aeruginosa* wild type (upper panels) or RpoN* (lower panels). All images taken on an Olympus stereoscope (mag. 4.5x). Scale bars are 100 μm. Points on the Kaplan-Meier survival curves represent the combined survival of three or more separate assays. Mantel-Cox log-rank tests performed to analyze curves (***p ≤ 0.0001).

A paralytic killing model, where lethal paralysis is mediated by hydrogen cyanide production by *P. aeruginosa*, was one of the infection assays used^37,40^. Some of the genes found to be associated with paralytic killing, particularly *hcnC, soxA, prpC*, and PA0745^40^, were affected by RpoN* in the microarray analysis. The test conditions were wild-type *P. aeruginosa* expressing RpoN* or the point mutant Y48A in RpoN* from an IPTG-inducible promoter. If RpoN* reduced gene expression of virulence factors, we expected to see increased *C. elegans* survival compared to the positive control. As expected, the positive control caused *C. elegans* paralysis and killing, while both negative controls had no effect on *C. elegans* survival (Fig. 6a). Expression of RpoN* or the Y48A point mutant significantly increased *C. elegans* survival compared to the wild type strain (Mantel Log Rank Test, p ≤ 0.0001). Furthermore, expression of RpoN* increased *C. elegans* survival significantly more compared to the Y48A point mutant (Mantel-Cox Log-Rank Test, p ≤ 0.0001). Thus, RpoN* expression improved worm survival in a *C*. *elegans–P*. *aeruginosa* infection model mediated by cyanide production.

### RpoN* prolongs *C. elegans* survival in a slow killing model

Multiple *P. aeruginosa–C*. *elegans* infection assays can be used to evaluate pathogenicity of the bacteria. The paralytic killing assay revealed that RpoN* improved *C*. *elegans* survival in an infection assay mediated by cyanide production. While the paralytic killing assay mimics infection conditions similar to a cystic fibrosis lung, other assays model alternate forms of *P. aeruginosa* infections^41^. Thus, we also evaluated the effects of RpoN* in a slow killing assay, where death is facilitated by the establishment and proliferation of an infection^38^, and is mediated by *lasR*, *gacA*, *lemA*, and *ptsP* ^38^. Wild-type *P. aeruginosa* PA19660 (empty vector) was the positive, virulent control. *P. aeruginosa ΔlasR*:*gent* ^*R*^ and *E. coli*, both lacking a vector, were negative, avirulent controls. The test condition was wild-type *P. aeruginosa* expressing RpoN* from an IPTG-inducible promoter. If RpoN* affected genes associated with *P. aeruginosa* pathogenicity, we expected improved *C*. *elegans* survival. As expected, the negative controls did not affect *C*. *elegans* survival (Fig. 6b). The positive control diminished *C*. *elegans* survival, with an LT_50_ of 72 h (Fig. 6b). *C*. *elegans* exposed to the wild-type strain appeared unhealthy as early as 48 h (Fig. 6c, top panels). RpoN* expression significantly improved *C*. *elegans* survival compared to wild type and increased the LT_50_ to 96 h (Fig. 6b; Mantel-Cox Log-Rank Test, p ≤ 0.0001). Furthermore, *C*. *elegans* appeared healthier on *P. aeruginosa* lawns expressing RpoN* (Fig. 6c, bottom panels). While it was expected that RpoN* would reduce *P. aeruginosa* virulence, there are several possible factors why RpoN* expression did not have a larger effect on *P. aeruginosa* survival in the slow killing assay. *C*. *elegans* often require higher concentrations of compounds due to selective uptake in the intestine and limited permeability of the cuticle^36^. In fact, a previous study reported that the levels of drug absorbed and metabolized by *C*. *elegans* was time and dose dependent, and that the presence of live bacteria decreased drug concentration in media^42^. Thus, it is possible that concentrations of gentamicin and IPTG were not sustained at adequate levels in the *C*. *elegans* gut to maintain selection and expression of the roadblock. A liquid assay would help alleviate this problem, as *C*. *elegans* would be suspended in media for the duration of the experiment^43^, which would allow gentamicin and IPTG to be pumped into the *C*. *elegans* gut as it feeds, helping to maintain selection and expression.

## Conclusion

Here, we demonstrated that the RpoN molecular roadblock altered gene expression and reduced virulence of *P. aeruginosa*. More genes were altered by RpoN* regulation than those with an RpoN consensus promoter, as the roadblock reduced expression of various regulatory proteins, subsequently reducing expression of their downstream targets. We also showed that in the absence of native RpoN, the molecular roadblock altered gene transcription, significantly reducing motility and other virulence-associated phenotypes. In these instances, RpoN* provides clues to the nuanced levels of transcriptional regulation within bacteria for genes controlled by multiple promoters. When native RpoN is absent, these interactions became clearer with notable decreases in gene expression of specific genes and reduction in specific virulence phenotypes. The molecular roadblock is a DNA binding agent that is cis-acting directly at RpoN consensus promoters. We propose that, regardless of the presence of native RpoN, the molecular roadblock alters transcription, reducing virulence in *P. aeruginosa* (Fig. 3c). Although mutations may arise to evade the molecular roadblock, the numerous RpoN binding sites within the *P. aeruginosa* genome precludes resistance. The effectiveness of the roadblock to reduce gene expression and virulence, along with the lower likelihood of developing resistance, suggests targeting RpoN consensus promoters is a powerful strategy to combat pathogenic *P. aeruginosa*.

Future studies will help us to further understand the effects and mechanism of action of the molecular roadblock. For instance, RpoN has been linked to *P. aeruginosa* tolerance of several antibiotics, specifically carbapenems, quinolones, and tobramycin^44–46^. The molecular roadblock reduced expression of *mex* family genes, including *mexH*, which are associated with multidrug efflux pumps. Additionally, the roadblock affected expression of *rpoS* and quorum sensing-related genes that regulate tolerance of *P. aeruginosa* to ofloxacin^47^. Thus, studies are needed to address synergistic effects between the roadblock and antibiotics. Additionally, the RpoN-box^25,27,48^, which facilitates DNA binding, as well as the RpoN consensus promoter sites^49,50^ are conserved among both Gram-positive and Gram-negative bacteria. Therefore, studies are also needed to evaluate potential broad-spectrum applications of the molecular roadblock. Unfortunately, the roadblock is not druggable in its current form, limiting its application, but its binding sites in the genome should serve as new targets for drug discovery. Finding small molecules with similar cis-acting function is crucial for development into a new antimicrobial. Targeting RpoN at its consensus promoters in a similar manner to the molecular roadblock may be an effective and desirable strategy to treat *P. aeruginosa* or other bacterial infections while minimizing the development of resistance.

## Materials and Methods

### Bacteria and Nematodes

*P. aeruginosa* PAO1 was provided by C. Manoil^37^ and D. Haas^16^. *P. aeruginosa* PA14 was provided by F. Ausubel^51^. *P. aeruginosa* PA19660 *Xen5* was purchased from PerkinElmer. The *lecA::luxΔlasR P. aeruginosa* PAO1 mutant was provided by S.P. Diggle^52^. The *P. aeruginosa* PAO1 Δ*rpoN* mutant was provided by D. Haas^16^. *E. coli* OP50 was provided by D. Pruyne (Upstate Medical University). Bacteria were grown overnight in Lennox Broth (Difco or Fisher BioReagents) at 37 °C with shaking, glycerol was added to 10%, and stocks were stored frozen at −80 °C. *Caenorhabditis elegans* N2 was obtained from the *Caenorhabditis* Genetics Center (University of Minnesota, Minneapolis, MN), and maintained on *E. coli* (OP50) on nematode growth media agar (NGM) at 20 °C^53^. To create a synchronized population, hermaphroditic, gravid adults were transferred by wire pick to fresh NGM plates with *E. coli* for 2–4 hours to lay eggs, and then removed. Eggs were grown to the young adult stage at 20 °C^54^.

### Plasmids

Plasmids and oligonucleotides used in this study are given in Supplementary Table S1. Plasmids were maintained in *E. coli* Top10 (Invitrogen). For plasmid and marker selection, the following antibiotics were used: ampicillin, 100 mg/L *E. coli*; kanamycin, 50 mg/L *E. coli* or 500 mg/L *P. aeruginosa*; gentamicin, 20 mg/L *E. coli* or 30 mg/L *P. aeruginosa*. For induction of gene expression, isopropyl β-D-1-thiogalactopyranoside (IPTG) was used, up to concentrations of 2 mM.

### Construction of RpoN*

A tightly regulated, IPTG-inducible expression vector for *P. aeruginosa* was constructed by cloning the region encoding LacI^Q^ and the *trc*-promoter of pTrc99a (Pharmacia Biotech, Sweden) into the *Xba* I and *Sph* I sites of the broad-host range plasmid pBBR1MCS-5 to yield pBRL320^55^. *EcoR* I and *Sac* I sites of the multiple cloning region in the pTrc99a fragment were removed by site-directed mutagenesis using Quikchange^TM^ (Stratagene) and oligonucleotides BL331.f/BL331.r (Supplementary Table S1) to give pBRL344.

A gene (rpoN*) encoding the last 60 amino acids of RpoN of *A. aeolicus* was synthesized and codon optimized for expression in *E. coli* by DNA 2.0 (Menlo Park, California). *Nde* I and *Sac* I restriction sites were engineered into the 5′ and 3′ ends of rpoN*, respectively. The rpoN* gene was then subcloned from pJ201:42178 into the *Nde* I/*Sac* I sites of the *E. coli* expression pET-vector pKH22 to give pBRL327^56^. pBRL327 was then digested with *Xba* I and *Sac* I to liberate the rpoN* gene with an upstream ribosome binding site. This fragment was cloned into the *Xba* I/*Sac* I sites of pBRL344 to yield pBRL348. To attenuate the rpoN* in pBRL348, the tyrosine at position 48 was changed to alanine using Quickchange^TM^ (Stratagene, Santa Clara, California) and oligonucleotides BL330.f/BL330.r to give pBRL349^34^.

### Transformation

Plasmids were introduced to *P. aeruginosa* through electroporation prior to all experiments, as previously described^57^. Transformed bacteria were selected for on brain heart infusion (BHI) or LB agar, and individual colonies were picked for each assay.

### RNA isolation and microarray analysis

RNA isolation and microarray analysis were done in quadruplicate. *P. aeruginosa* PAO1 or *P. aeruginosa ΔrpoN* were transformed with either pBRL344 or pBRL348 were grown in LB supplemented with 30 mg/L gentamicin for 24 h at 37 °C and 200 rpm. Fresh LB supplemented with 30 mg/L gentamicin was inoculated with 0.5% (v/v) of the LB-grown seed culture. The inoculated cultures were then grown at 37 °C and 200 rpm to an optical density at 600 nm (OD_600_) of 0.2. Cultures were treated with IPTG at a final concentration of 1 mM, and induced cultures were grown for an additional 2 h. For *P. aeruginosa ΔrpoN*, media was supplemented with 1 mM glutamine. RNA was purified using a Qiagen RNeasy kit with addition of RNAprotect® reagent and on-column DNase digestion^58,59^. RNA samples were analyzed for quality with a Bioanalyzer (Agilent) and for DNA contamination by PCR. Microarray studies were carried out at the Microarray Core Facility at SUNY Upstate Medical University (Syracuse, NY). Microarray experiments were performed as indicated in the Affymetrix GeneChip® Expression Analysis Technical Manual (Pub. 702232, Rev. 3) and by established protocol^60^. Data was processed with Affymetrix software for quality control, calculating signal intensities, and indicating presence of a gene. The RMA method was used to normalize data (GeneTraffic software, Stratagene, La Jolla). Additional statistical analysis was performed with the MultiExperiment Viewer (MeV v4.6.2) to identify genes with significant differences in intensities. Microarray data was deposited in Gene Expression Omnibus and is accessible through GEO Series accession number GSE35632^61^.

### Phenotyping Assays

Assays to measure swimming and twitching^62^, protease^63^, elastase^64,65^, pyoverdine and pyocyanin^66^, and rhamnolipids^67,68^ were conducted according to standard protocols. Freshly transformed *P. aeruginosa* PA19660 *Xen5,* ﻿PAO1﻿ wild type,or PAO1 *ΔrpoN*, and *lecA::luxΔlasR P. aeruginosa* PAO1 were grown on the appropriate media with 30 µg/mL gentamicin with or without 2 mM IPTG. Plates for motility, protease and elastase assays were incubated for 24–48 h at 37 °C and photographs were taken and bioluminescence was measured by IVIS (PerkinElmer). For the motility assays, the colony diameter was measured across the point of inoculation to the edges of the bacterial patch using Living Image software (PerkinElmer). Elastase, pyoverdine and pyocyanin assays in liquid broth were grown at 37 °C, 200 rpm for the appropriate time and absorbance measured at 459 nm, 408 nm, and 520 nm, respectively. For PAO1 and PA19660, media was not supplemented with additional nutrients as it was unnecessary for adequate growth. For the Δ*rpoN* mutant, media was supplemented with 1 mM glutamine.

### Biofilm Assay

Freshly transformed *P. aeruginosa* PA14 were inoculated in LB broth with 30 mg/L gentamicin and grown overnight at 37 °C. Cultures were diluted 1:3 in LB containing 30 mg/L gentamicin and 1 mM IPTG, grown for 3 h at 37 °C, and subcultured 1:50 in M63 minimal media with 0.4% arginine, 1 mM MgSO_4_, 30 mg/L gentamicin, and 1 mM IPTG. Air-liquid interface (ALI) and microtiter dish biofilm formation assays were conducted by standard protocol^69–71^. For the ALI assay, biofilms were visualized by phase contrast at 200x on a Leica AF6000 microscope. For the microtiter dish assay, biofilms were stained with crystal violet, extracted in ethanol and absorbance at 550 nm was measured on a Synergy H1 Multi-Mode reader (Biotek).

### Paralytic Killing Assay

Freshly transformed *P. aeruginosa* PAO1 were swabbed onto BHI agar with 30 mg/L gentamicin and at least 1 mM IPTG, when appropriate. For *lecA::luxΔlasR P. aeruginosa* PAO1 and *E. coli* (OP50), bacteria were grown from frozen stocks overnight at 37 °C with shaking in BHI broth containing or lacking 30 mg/L gentamicin, respectively. These overnight cultures were diluted 1:100 in BHI and 170 μL was spread on BHI agar with or without antibiotics, per standard protocol^37^. All BHI agar plates contained 1.7% BactoAgar in 60-mm petri plates. Plates were incubated at 37 °C for 24 h.

### Slow Killing Assay

Freshly transformed *P. aeruginosa* PA19660 *Xen5* was swabbed onto modified NGM (0.35% peptone)^38^ with 30 mg/L gentamicin and when applicable, at least 1 mM IPTG. For *lecA::luxΔlasR P. aeruginosa* PAO1 and *E. coli* (OP50), fresh bacteria plates were used to inoculate LB with or without 30 mg/L gentamicin, respectively, and grown overnight at 37 °C with shaking. A 30 µL volume of overnight culture was spread on modified NGM with or without antibiotics, per standard protocol^38^. All modified NGM plates were incubated at 37 °C for 24 h, then at room temperature (22–26 °C) for an additional 24 h.

### Statistics

Data were analyzed using Excel and GraphPad Prism with a significance of *p* ≤ 0.05 (Microsoft, Washington; GraphPad Software Inc., California).

### Data Availability

The datasets generated during the current study are deposited in the Gene Expression Omnibus and is accessible through GEO Series accession number GSE35632 (https://www.ncbi.nlm.nih.gov/geo/query/acc.cgi?acc=GSE35632). Otherwise, the datasets generated for and analyzed during the current study are either included in this article (and its supplementary files) or is available from the corresponding author on reasonable request.

## Electronic supplementary material

Supplementary Tables

Supplementary Figures

Supplementary Note
