# Supplementary material for: Targeting the alternative sigma factor RpoN to combat virulence in Pseudomonas aeruginosa
Source: Sci Rep. 2017 Oct 3;7:12615. doi: 10.1038/s41598-017-12667-y (PMC5626770; doi:10.1038/s41598-017-12667-y)
Supplement: Supplementary file 1 — Supplementary Tables [file 41598_2017_12667_MOESM1_ESM.pdf]

Targeting the alternative sigma factor RpoN to combat virulence in *Pseudomonas aeruginosa*

Megan G. Lloyd[1]; Benjamin R. Lundgren[2,3]; Clayton W. Hall[4]; Luke B.-P. Gagnon[4]; Thien-Fah Mah[4]; Jennifer F. Moffat[1]; Christopher T. Nomura[2,3*]

[1]Department of Microbiology and Immunology, SUNY Upstate Medical University, Syracuse, NY, USA

[2]Department of Chemistry, SUNY College of Environmental Science and Forestry, Syracuse, NY, USA

[3]Center for Applied Microbiology, SUNY College of Environmental Science and Forestry, Syracuse, NY, USA

[4]Department of Biochemistry, Microbiology, and Immunology, University of Ottawa, Ottawa, Ontario, Canada

* Corresponding author

E-mail: ctnomura@esf.edu

Supplemental Table 1. Plasmids and Oligonucleotides used in this study. Abbreviations for antibiotic selection: ampicillin (AmR), kanamycin (KmR), and gentamicin (GmR). Oligonucleotides (5' to 3') were purchased from Integrated DNA Technologies (Iowa, U.S.A.).

Plasmids	Description	Source
pBBR1MCS-5	Broad-host range cloning vector; Gm^R	-38
pTrc99a	General cloning expression vector; Am^R	Pharmacia
pKH22	pET-derived expression vector; Am^R	-39
pJ201:42178	pUC ori, Km^R, codon optimized synthetic RpoN region III from A. aerolicus	DNA 2.0
pBRL320	$lacI^Q$-trc promoter of pTrc99a in pBBR1MCS5; Gm^R	This study
pBRL327	codon optimized synthetic RpoN region III from A. aerolicus in pKH22; Am^R	This study
pBRL344	same as pBRL320 but both EcoR I and Sac I restriction sites are absent from the MCS region of cloned pTrc99a fragment; Gm^R	This study
pBRL348	pBRL344, codon optimized synthetic RpoN region III from A. aerolicus	This study
pBRL349	pBRL348, Y48A RpoN (synthetic)	This study
**Oligonucleotide**	**Sequence**	
BL330.f	cgtacggttgccaaggctcgtgagatgctgggtattccg	
BL330.r	cggaatacccagcatctcacgagccttggcaaccgtacg	
BL331.f	ggaaacagaccatgcaattccagctcggtacccggggatcc	
BL331.r	ggatccccgggtaccgagctggaattgcatggtctgtttcc	
BL343.f	ttaggcctgaatgccggtg	
BL343.r	atgtacgcagtgattgttaccg	

Supplemental Table 2. Genes in *Pseudomonas aeruginosa* PAO1 with a 2-fold or greater change in transcription when RpoN* molecular roadblock is expressed. Complete data available at GEO accession: GSE35632.

PA Gene ID:	Gene Name:	Fold Change
PA4825	*mtgA*	-88.95477
PA4217	*phzS*	-76.10926
PA4211	*phzB1*	-62.90052
PA4823		-59.40452
PA3724	*lasB*	-41.42702
PA4824		-36.69471
PA0200		-33.30115
PA5220		-23.18307
PA1905	*phzG2*	-22.12339
PA4129		-21.33276
PA0049		-21.07557
PA5348		-19.39348
PA1901	*phzC2*	-18.50701
PA3331		-17.66104
PA2381		-16.91230
PA1903	*phzE2*	-16.85378
PA2114		-16.42134
PA4209	*phzM*	-16.11129
PA1904	*phzF2*	-15.94464
PA1902	*phzD2*	-15.75240
PA4133		-15.69792
PA2570	*pa1L*	-15.69790
PA4130		-14.87688
PA4500		-14.64666
PA1871	*lasA*	-14.44502
PA4131		-13.95297
PA1556		-13.57142
PA5172	*arcB*	-12.66258
PA2939		-12.10474
PA0865	*hpd*	-12.08378
PA1342		-11.83510
PA3334		-11.73300
PA0852	*cpbD*	-11.61165
PA3361		-11.59154

PA4739		-11.37268
PA2445	*gcvP2*	-11.08088
PA3187		-10.68489
PA3126	*ibpA*	-10.61108
PA2604		-10.61108
PA3049	*rmf*	-10.28521
PA4141		-9.76419
PA3186	*oprB*	-9.74728
PA3332		-9.64646
PA2112		-9.43159
PA2300	*chiC*	-9.35022
PA0132		-9.33403
PA0122		-9.31787
PA3328		-9.12611
PA2001	*atoB*	-9.00047
PA2008	*fahA*	-8.84584
PA2111		-8.75435
PA2444	*glyA2*	-8.72406
PA3333	*fabH2*	-8.57418
PA5173	*arcC*	-8.45615
PA2000	*dhcB*	-8.29650
PA3337	*rfaD*	-8.18226
PA2007	*maiA*	-8.00000
PA2247	*bkdA1*	-8.00000
PA4822		-7.75431
PA3330		-7.54228
PA4502		-7.46426
PA2248	*bkdA2*	-7.45134
PA1555	*ccoP2*	-7.36150
PA0546	*metK*	-7.31065
PA1337	*ansB*	-7.29800
PA3190		-7.22251
PA4236	*katA*	-7.22251
PA3329		-7.12307
PA3691		-6.97648
PA2113	*opdO*	-6.86852
PA3335		-6.84476
PA4501	*opdP*	-6.84476
PA4607		-6.78572
PA3922		-6.76224
PA4296	*pprB*	-6.71552
PA1557	*ccoN2*	-6.69229

PA3569	*mmsB*	-6.68070
PA2119		-6.63456
PA3785		-6.62307
PA3327		-6.60016
PA2553		-6.60016
PA0459		-6.53189
PA3923		-6.49802
PA2193	*hcnA*	-6.43081
PA4132		-6.40856
PA1914		-6.39746
PA3570	*mmsA*	-6.38639
PA3692	*lptF*	-6.26581
PA5415		-6.26581
PA3188		-6.25496
PA4306	*flp*	-6.25496
PA1673		-6.07338
PA2069		-6.06287
PA1984	*exaC*	-5.98977
PA0587		-5.96904
PA2009	*hmgA*	-5.84620
PA5421	*fdhA*	-5.84620
PA1999	*dhcA*	-5.84620
PA5171	*arcA*	-5.66666
PA4206	*mexH*	-5.63728
PA4496		-5.51171
PA4587	*ccpR*	-5.44526
PA5167		-5.38893
PA2443	*sdaA*	-5.36100
PA4205	*mexG*	-5.33319
PA5153		-5.32396
PA4134		-5.26889
PA4922	*azu*	-5.26889
PA0266	*gabT*	-5.24157
PA4067	*oprG*	-5.22344
PA2250	*lpdV*	-5.21440
PA3723		-5.18736
PA2110		-5.17838
PA3479	*rhlA*	-5.17838
PA2116		-5.13370
PA5054	*hslU*	-5.12482
PA0085	*hcp1*	-5.10709
PA2194	*hcnB*	-5.09824

PA1340		-5.06303
PA0588		-5.05426
PA0586		-5.01066
PA4590	*pra*	-4.97605
PA4542	*clpB*	-4.96743
PA2249	*bkdB*	-4.95025
PA3478	*rhlB*	-4.91605
PA1341		-4.89904
PA2573		-4.85678
PA0130		-4.78162
PA3792	*leuA*	-4.74859
PA0139	*ahpC*	-4.71579
PA4210		-4.66701
PA2446	*gcvH2*	-4.61075
PA1562	*acnA*	-4.55516
PA1074	*braC*	-4.54727
PA2575		-4.52369
PA2195	*hcnC*	-4.43828
PA2554		-4.41527
PA3622	*rpoS*	-4.41526
PA2552		-4.40762
PA2003	*bdhA*	-4.39999
PA1939		-4.36203
PA0871	*phhB*	-4.36203
PA4714		-4.33941
PA0872	*phhA*	-4.32440
PA4463		-4.32440
PA4880		-4.30944
PA4625	*cdrA*	-4.27226
PA0084	*tssC1*	-4.27226
PA0910		-4.23541
PA5427	*adhA*	-4.22075
PA2031		-4.20615
PA5027		-4.18434
PA3477	*rhlR*	-4.16264
PA4761	*dnaK*	-4.15544
PA2805		-4.09823
PA1930		-4.09113
PA3831	*pepA*	-4.07698
PA0870	*phhC*	-4.06992
PA1339		-3.97926
PA5475		-3.97926

PA2014	*liuB*	-3.97237
PA0107		-3.95863
PA2513	*antB*	-3.95863
PA1874		-3.93810
PA5369	*pstS*	-3.92448
PA3195	*gapA*	-3.87716
PA3040		-3.87045
PA1789		-3.86375
PA0083	*tssB1*	-3.79737
PA1324		-3.78423
PA4352		-3.77768
PA1344		-3.76461
PA2815		-3.73861
PA0905	*rsmA*	-3.73860
PA0269		-3.73213
PA0779		-3.72567
PA4506	*dppF*	-3.72567
PA4503		-3.71278
PA3326	*clpP2*	-3.70635
PA3712		-3.66167
PA1323		-3.65533
PA0106	*coxA*	-3.64900
PA0911		-3.64900
PA1596	*htpG*	-3.62379
PA5154		-3.61752
PA3531	*bfrB*	-3.61125
PA3021		-3.59253
PA0300	*spuD*	-3.56771
PA1888		-3.54307
PA0299	*spuC*	-3.49429
PA0745		-3.48220
PA5355	*glcD*	-3.45216
PA0985	*pyoS5*	-3.44618
PA2274		-3.43426
PA0315		-3.41054
PA3309		-3.40464
PA2442	*gcvT2*	-3.40463
PA3189		-3.39286
PA1135		-3.38698
PA1869		-3.36942
PA5112	*estA*	-3.36942
PA0048		-3.35776

PA5053	*hslV*	-3.34035
PA1041		-3.32303
PA2754		-3.32303
PA2232	*pslB*	-3.29436
PA5178		-3.29436
PA1579		-3.28866
PA3031		-3.27728
PA0050		-3.26594
PA2012	*liuD*	-3.26029
PA2067		-3.24901
PA4611		-3.24338
PA4504		-3.20984
PA5253	*algP*	-3.19873
PA2013	*liuC*	-3.19873
PA4385	*groEL*	-3.18766
PA2753		-3.18215
PA3784		-3.17664
PA4497		-3.15469
PA1338	*ggt*	-3.14923
PA0807	*ampDh3*	-3.13834
PA3688		-3.13290
PA4577		-3.12748
PA4876	*osmE*	-3.12206
PA0918		-3.10588
PA4015		-3.10050
PA4738		-3.06843
PA4614	*mscL*	-3.05781
PA1818	*ldcA*	-3.05252
PA0131		-3.05252
PA5352		-3.05252
PA3418	*ldh*	-3.04196
PA0795	*prpC*	-3.04196
PA5100	*hutU*	-3.04196
PA1244		-3.04196
PA2788		-3.02618
PA2015	*liuA*	-3.01049
PA0656		-3.00528
PA4842		-3.00008
PA5546		-2.99488
PA0907		-2.99488
PA1216		-2.98452
PA0542		-2.97936

PA0866	*aroP2*	-2.97936
PA0265	*gabD*	-2.96905
PA2622	*cspD*	-2.94854
PA4386	*groES*	-2.93325
PA0867	*mliC*	-2.93325
PA0298	*spuB*	-2.92817
PA1551		-2.92817
PA0070	*tagQ1*	-2.92310
PA5168		-2.92310
PA0836	*ackA*	-2.90291
PA1617		-2.89287
PA3520		-2.89287
ig_517462_518083		-2.86791
PA5397		-2.85799
PA4760	*dnaJ*	-2.84317
PA3572		-2.83825
PA1803	*lon*	-2.82353
PA3041		-2.82353
PA5191		-2.81864
PA4826		-2.80889
PA4026		-2.80403
PA2577		-2.79917
PA5419	*soxG*	-2.79917
PA5181		-2.79433
Pae_tRNA_Arg_s		-2.78949
PA0743		-2.78949
PA1432	*lasI*	-2.78949
PA3182	*pgl*	-2.75108
PA0744		-2.73682
PA5417	*soxD*	-2.72735
PA0473		-2.72263
PA4759	*dapB*	-2.72263
PA1592		-2.71791
PA5418	*soxA*	-2.71321
PA0908		-2.69914
PA0447	*gcdH*	-2.69447
PA1657		-2.69447
PA1493	*cysP*	-2.68050
PA2501		-2.67122
ig_4713795_4713098		-2.66660
PA2197		-2.66198
PA1658		-2.65737

PA1784		-2.64818
PA1429		-2.64359
PA1847		-2.64359
PA3417		-2.62989
PA5545		-2.62533
PA3614		-2.62533
PA2915		-2.60720
PA2024		-2.60268
PA1080	*flgE*	-2.59368
PA4525	*pilA*	-2.58023
PA0108	*coIII*	-2.57576
PA0808		-2.56241
PA4733	*acsB*	-2.56241
PA1546	*hemN*	-2.55797
PA3042		-2.54471
PA2433		-2.54030
PA3336		-2.54030
PA4328		-2.51403
PA2698		-2.51403
PA4608		-2.51403
PA5312		-2.50533
PA3832	*holC*	-2.50533
PA4874		-2.49666
PA4762	*grpE*	-2.49666
PA1677		-2.49234
PA3666	*dapD*	-2.48802
PA0195	*pntA*	-2.48372
PA4648		-2.48372
PA3236		-2.47941
PA0746		-2.47512
PA1174	*napA*	-2.46656
PA4139		-2.45803
PA0301	*spuE*	-2.45377
PA5119	*glnA*	-2.44105
PA3972		-2.43682
PA5208		-2.43260
PA0302	*spuF*	-2.41580
PA0038		-2.40327
PA4207	*mexI*	-2.39911
PA4208	*ompD*	-2.39496
PA5245		-2.39496
PA3613		-2.39081

PA2177		-2.37841
PA0467		-2.37430
PA4498		-2.37430
PA2760	*oprQ*	-2.37430
PA5528		-2.37019
PA3753		-2.36608
PA0506		-2.35790
PA2512	*antA*	-2.35790
PA1967		-2.35790
PA1196		-2.34974
PA2071	*fusA2*	-2.34161
PA3250		-2.33351
PA5366	*pstB*	-2.31739
PA4311		-2.30537
PA0304	*spuH*	-2.30138
PA5360	*phoB*	-2.30138
PA2066		-2.29740
PA4624	*cdrB*	-2.29342
PA1288		-2.28548
PA3931		-2.28153
PA3385	*amrZ*	-2.27758
PA4031	*ppa*	-2.27758
PA0250		-2.27363
PA0634		-2.26970
PA0854	*fumC2*	-2.26577
PA2562		-2.26577
PA3879	*narL*	-2.25793
PA0547		-2.25402
PA1094	*fliD*	-2.25012
PA0622		-2.24622
PA0960		-2.24622
PA5219		-2.24233
PA0615		-2.24233
PA4651		-2.23845
PA2223		-2.23845
PA3819		-2.23071
PA5396		-2.20381
PA4505		-2.19999
PA1544	*anr*	-2.19619
PA5098	*hutH*	-2.19619
PA0090	*clpV1*	-2.18859
PA4204	*ppgL*	-2.18859

PA3501		-2.18102
PA2068		-2.16971
PA1940		-2.16971
PA1071	*braF*	-2.16595
PA5313		-2.15846
PA1604		-2.15846
PA0176	*aer2*	-2.15099
PA0114	*senC*	-2.15099
PA5380	*gbdR*	-2.15099
PA4610		-2.13984
PA5314		-2.13613
PA0624		-2.12874
PA2365		-2.12506
PA2030		-2.12138
PA1746		-2.11770
PA2826		-2.10672
PA3415		-2.09943
PA1571		-2.09943
PA5169		-2.09580
PA2378		-2.09217
PA5543		-2.09217
PA0140	*ahpF*	-2.08855
PA2555		-2.08493
PA5354	*glcE*	-2.08132
PA1327		-2.08132
PA4735		-2.08132
PA0626		-2.08132
PA0446		-2.07772
PA4175	*piv*	-2.07412
PA3848		-2.07412
PA5271		-2.07412
PA3529		-2.06337
PA0815		-2.06337
PA1217		-2.06337
PA3181		-2.06337
PA0835	*pta*	-2.05623
PA4913		-2.05623
PA0201		-2.05267
PA0126		-2.04911
PA0853		-2.04911
PA0423	*pasP*	-2.04557
PA3615		-2.04557

PA0111		-2.04202
PA5060	*phaF*	-2.04202
PA2620	*clpA*	-2.03849
PA5364		-2.03496
PA1941		-2.03144
PA2776		-2.02792
PA0962		-2.02441
PA1073	*braD*	-2.02090
PA5372	*betA*	-2.02090
PA0637		-2.01391
PA1880		-2.01391
PA1326	*ilvA2*	-2.00694
PA5058	*phaC2*	-2.00694
PA3440		-2.00347
PA3014	*faoA*	-2.00347
PA1777	*oprF*	-2.00000
PA1564		2.00000
PA3806		2.00000
PA3242		2.00347
PA2742	*rpmI*	2.01740
PA3979		2.01740
PA5216		2.01740
PA1195		2.02441
PA2020		2.02441
PA2950		2.02792
PA1776	*sigX*	2.02792
PA4664	*hemK*	2.02792
PA1800	*tig*	2.03144
PA3169	*mtnA*	2.03849
ig_6125795_6125079		2.04202
Pae_tRNA_Trp_f		2.04202
PA4051	*thiL*	2.04557
PA3646	*lpxD*	2.04557
PA4450	*murA*	2.05623
PA5117	*typA*	2.05979
PA0922		2.05980
PA3211		2.06337
PA0802		2.06337
PA3983		2.06337
PA4007	*proA*	2.06694
PA2772		2.06695
PA4566	*obg*	2.06695

PA1037		2.07053
PA1965		2.07053
PA5414		2.07772
PA2384		2.08493
PA5336	*gmk*	2.08855
PA0966	*ruvA*	2.08855
PA4003	*pbpA*	2.09943
PA1714	*exsD*	2.09943
PA1716	*pscC*	2.11038
PA4050	*pgpA*	2.11038
PA3657	*map*	2.11038
PA0168		2.11770
PA2733		2.11770
PA1044		2.12138
PA5503		2.12874
PA3654	*pyrH*	2.13243
PA1199		2.13243
PA1964		2.13243
PA5563	*soj*	2.13613
ig_3545880_3545073		2.13613
PA5332	*crc*	2.13613
PA0595	*ostA*	2.13984
PA0608		2.14355
PA1457	*cheZ*	2.14726
PA1741		2.14726
PA3981		2.14726
PA4482	*gatC*	2.14726
PA5560	*atpB*	2.14726
PA3828		2.15099
PA4782		2.15099
PA0968		2.15846
PA1452	*flhA*	2.16220
PA1712	*exsB*	2.17347
PA5465		2.17347
PA0017		2.18102
PA2741	*rplT*	2.18102
PA2860		2.18102
PA0805		2.18480
PA0668	*tyrZ*	2.18859
PA3129		2.20381
PA0068		2.20763
PA1305		2.21146

PA3903	*prfC*	2.21146
PA5002		2.21530
PA4846	*aroQ1*	2.22299
PA3881		2.22299
PA0733		2.22299
PA4698		2.22684
PA1117		2.22684
PA1453	*flhF*	2.23071
PA3109		2.23071
PA3110		2.23845
PA4226	*pchE*	2.24622
ig_2558918_2557964_s		2.25012
PA0659		2.25402
PA5001		2.25793
PA2774		2.26184
PA1722	*pscI*	2.26577
PA4765	*omlA*	2.26577
PA0729		2.26970
PA2453		2.27758
PA4847	*accB*	2.27758
PA0723	*coaB*	2.28548
PA2975	*rluC*	2.29342
PA4267	*rpsG*	2.29740
PA3650	*dxr*	2.29740
PA1394		2.30537
PA0965	*ruvC*	2.30537
Pae_tRNA_Ala_f		2.31338
PA1200		2.31338
PA5274	*rnk*	2.31739
PA2969	*plsX*	2.32543
PA1475	*ccmA*	2.32947
PA3815	*iscR*	2.33351
PA4996	*rfaE*	2.34161
PA4852		2.34161
PA3620	*mutS*	2.34161
PA0592	*ksgA*	2.34567
PA5338	*spoT*	2.37019
PA3741		2.37430
PA3807	*ndk*	2.38254
PA1098	*fleS*	2.38254
PA4116	*bphO*	2.39081
PA5504		2.39081

PA4135		2.39496
PA4266	*fusA1*	2.39496
Pae_tRNA_Leu_s		2.39496
PA1100	*fliE*	2.40327
PA4479	*mreD*	2.40327
PA4997	*msbA*	2.40744
PA5564	*gidB*	2.41580
PA4449	*hisG*	2.41999
PA1582	*sdhD*	2.42419
PA3719	*armR*	2.43260
PA3111	*folC*	2.43260
PA4379		2.43682
PA1773	*cmaX*	2.43682
PA3009		2.43682
PA4271	*rplL*	2.45377
PA1192		2.46229
PA2679		2.47084
PA0719		2.48802
PA4700	*mrcB*	2.49234
PA3578		2.49666
PA4238	*rpoA*	2.49666
PA4043	*ispA*	2.49666
PA4635		2.50967
PA2629	*purB*	2.51839
PA4481	*mreB*	2.52275
PA5445		2.52276
PA4629		2.53590
ig_3527428_3526677		2.54912
PA4006	*nadD*	2.55797
PA3144		2.55797
PA3655	*tsf*	2.56685
Pae_tRNA_Gln_s		2.57130
PA3152	*hisH2*	2.57130
ig_1117390_1118158		2.58023
PA3151	*hisF2*	2.58470
PA4636		2.58919
PA3142		2.58919
PA4004		2.59368
PA2973		2.59818
PA2288		2.60268
PA4323		2.60268
PA3266	*capB*	2.60268

PA1812	*mltD*	2.60268
PA4237	*rplQ*	2.60720
PA1031		2.61172
PA4255	*rpmC*	2.61625
PA5337	*rpoZ*	2.62988
PA0722		2.62989
PA3178		2.63902
PA5565	*gidA*	2.65277
PA1610	*fabA*	2.65737
PA1711	*exsE*	2.65737
PA3148	*wbpI*	2.66198
PA4480	*mreC*	2.66198
PA4671		2.67122
PA2967	*fabG*	2.68050
PA1317	*cyoA*	2.70851
PA4251	*rplE*	2.70851
PA3577		2.74157
PA4853	*fis*	2.75108
PA5174		2.76542
PA4672		2.77984
PA4810	*fdnI*	2.77984
PA3162	*rpsA*	2.77984
PA1581	*sdhC*	2.80403
PA4742	*truB*	2.80403
PA4325		2.80889
PA4069		2.83825
PA4239	*rpsD*	2.84317
PA3154	*wzy*	2.86295
PA4745	*nusA*	2.86295
Pae_tRNA_Phe_f		2.86295
PA2460		2.88286
PA4665	*prfA*	2.88786
PA0718		2.90291
PA4250	*rpsN*	2.90291
PA0128		2.91804
PA4240	*rpsK*	2.93834
PA4743	*rbfA*	2.94854
PA4662	*murI*	2.96391
PA2974		2.96905
PA5340		2.98452
PA4247	*rplR*	2.98970
PA3179		2.99488

PA4451		2.99488
Pae_tRNA_Ile_f		3.00528
PA3644	*lpxA*	3.02095
PA3353		3.02618
PA4748	*tpiA*	3.04196
PA3145	*wbpL*	3.05252
PA4115		3.06312
PA4042	*xseB*	3.06843
PA4567	*rpmA*	3.07375
PA5118	*thiI*	3.08977
PA0354		3.09513
PA4640	*mqoB*	3.12206
PA3645	*fabZ*	3.17664
PA4268	*rpsL*	3.17664
PA1775	*cmpX*	3.18215
PA1281	*cobV*	3.18215
PA3526		3.19319
PA3616		3.22098
PA5561	*atpI*	3.23216
PA3643	*lpxB*	3.23216
PA1240		3.24338
PA3656	*rpsB*	3.24901
PA1105	*fliJ*	3.26029
PA4568	*rplU*	3.26594
PA3818		3.30008
PA4940		3.32303
PA4241	*rpsM*	3.32303
PA1852		3.34615
PA4256	*rplP*	3.35776
PA4258	*rplV*	3.36359
PA5339		3.38112
PA4741	*rpsO*	3.38698
PA4248	*rplF*	3.38698
ig_1947041_1948502		3.39285
PA2019		3.39285
PA5570	*rpmH*	3.39874
PA4274	*rplK*	3.43426
PA4275	*nusG*	3.47016
PA4260	*rplB*	3.47617
PA2968	*fabD*	3.47617
PA4244	*rplO*	3.50642
PA2840		3.51250

PA4259	*rpsS*	3.56772
PA4254	*rpsQ*	3.57390
PA4252	*rplX*	3.58010
PA4253	*rplN*	3.59876
PA4242	*rpmJ*	3.64900
PA4243	*secY*	3.65533
PA5049	*rpmE*	3.65533
PA1639		3.67438
PA4932	*rplI*	3.69994
PA0577	*dnaG*	3.69994
PA4245	*rpmD*	3.70635
PA4273	*rplA*	3.70635
PA3246	*rluA*	3.74509
ig_5207621_5208463		3.79079
PA4220		3.80395
PA4249	*rpsH*	3.81055
PA4670	*prs*	3.81055
PA4246	*rpsE*	3.82378
PA4934	*rpsR*	3.84371
Pae_tRNA_Gly_s		4.02085
PA4276	*secE*	4.02085
PA4433	*rplM*	4.03481
PA5569	*rnpA*	4.04881
PA3149	*wbpH*	4.07698
PA4432	*rpsI*	4.08405
PA0758		4.08405
PA4257	*rpsC*	4.11246
Pae_tRNA_Tyr_s		4.12673
PA3982		4.14106
PA4746		4.18434
PA2971		4.19887
PA4935	*rpsF*	4.21344
PA4753		4.21344
PA5315	*rpmG*	4.22807
PA2970	*rpmF*	4.53153
PA3146	*wbpK*	4.57097
PA3745	*rpsP*	4.66701
PA4933		4.91605
PA4272	*rplJ*	5.04551
PA0579	*rpsU*	5.07181
PA3744	*rimM*	5.10708
PA4263	*rplC*	5.11594

PA3742	*rplS*	5.14261
PA4563	*rpsT*	5.15153
PA0578		5.17838
PA2584	*pgsA*	5.24157
PA4262	*rplD*	5.36099
PA4261	*rplW*	5.37029
PA5568		5.52127
PA4264	*rpsJ*	5.68633
PA5316	*rpmB*	5.94839
PA5471		6.15816
PA3743	*trmD*	6.43081
PA3147	*wbpJ*	6.48677
PA3642	*rnhB*	6.58873
PA5470		6.86852
PA0734		8.05564
PA1718	*pscE*	13.61854

Supplementary Table S3. List of genes affected by loss of native RpoN or expression of RpoN* in *Pseudomonas aeruginosa*. Select genes with altered transcription. Comparisons include: *P. aeruginosa* PAO1 and the *P. aeruginosa ΔrpoN* strain each harboring the empty vector plasmid (E.V.); and *P. aeruginosa ΔrpoN* strain with the empty vector plasmid and *ΔrpoN* strain expressing RpoN*. *P. aeruginosa* PAO1 grown in LB broth, and *P. aeruginosa ΔrpoN* grown in LB broth supplemented with 1 mM glutamine. Mark "---" indicates no change in transcription.

Regulator	Gene ID	Function	PAO1 - EV v. ∆RpoN - EV	∆RpoN - EV v. ∆RpoN - RpoN*
RpoN	PA2445	Glycine cleavage system protein P2	-15.6	---
RpoN, FecI	PA1342	Amino acid transport	-11.0	-2.9
RpoN	PA1094	*fliD*, flagellar capping protein	-10.3	---
RpoN, AlgU	PA3692	*lptF*, lipotoxon F	-9.0	---
RpoN	PA2604	Conserved hypothetical protein, localized to membrane	-6.7	-2.1
RpoN	PA2446	Glycine cleavage system protein H2	-5.3	---
RpoN (RpoD)	PA4463	Conserved hypothetical protein, localized to cytoplasm	-4.5	-2.7
RpoN (RpoD)	PA4590	*pra*, protein activator	-4.3	---
RpoN	PA5119	*glnA*, glutamine synthetase	-3.0	-1.4
RpoN	PA4726	*cbrB*, two-component response regulator	---	-1.4
RpoN	PA2740	*pheS*, phenylalanyl-tRNA ligase subunit alpha	---	-2.0
**RpoN, RpoS**	**PA3477**	***rhlR*, transcriptional regulator**	**---**	**-2.8**
RhlR	PA4217	*phzS*, flavin-containing monooxygenase	-43.0	-1.6
	PA3724	*lasB*, elastase	-13.9	-3.2
	PA1871	*lasA*, elastase	-10.7	-1.3
	PA2009	*hmgA*, homogentisate 1,2-dioxygenase	-10.5	-2.3
	PA4211	*phzB1*, probable phenazine biosynthesis protein	-10.4	-9.2
	PA4209	*phzM*, probable phenazine-specific methyltransferase	-7.9	-1.5
	PA1901	*phzC2*, phenazine biosynthesis protein	-7.7	-3.2
	PA4296	*pprB*, biofilm regulator	-6.1	-1.3
	PA0852	*cbpD*, chitin-binding protein	-5.5	-1.1
	PA1902	*phzD2*, phenazine biosynthesis protein	-5.0	-2.8
	PA1903	*phzE2*, phenazine biosynthesis protein	-4.7	-4.1
	PA1905	*phzG2*, probable pyridoxamine 5'-phosphate oxidase	-4.5	-4.9
	PA1904	*phzF2*, probable phenazine biosynthesis protein	-4.0	-4.0
	PA2193	*hcnA*, hydrogen cyanide synthase	-3.3	-1.9
	PA1596	*htpG*, heat shock protein	-3.3	-1.3
	PA3478	*rhlB*, rhamnosyltransferase chain B	-3.1	-1.4
	PA2194	*hcnB*, hydrogen cyanide synthase	-2.7	-1.8
	PA2195	*hcnC*, hydrogen cyanide synthase	-2.3	-1.6
**RpoN**	**PA1544**	***anr*, transcriptional regulator**	**---**	**-2.1**
Anr	PA5172	*arcB*, arginine metabolism	-20.6	-1.7
	PA5171	*arcA*, arginine metabolism	-12.4	-1.8
	PA5170	*arcD*, arginine metabolism	-10.1	-2.3

	PA3337	*rfaD*, LPS-core related	-8.8	-1.4
	PA4067	*oprG*, outer membrane protein	-7.2	-3.4
	PA1557	*ccoN2*, cytochrome c oxidase	-5.2	-1.5
	PA0459	ClpA/B protease ATP binding subunit	-3.1	-2.1
**RpoN**	**PA0652**	***vfr*, transcriptional regulator**	**---**	**-1.5**
Vfr	PA4552	*pilW*, type 4 fimbrial biogenesis protein	---	-1.7
