# Supplementary material for: Targeting the alternative sigma factor RpoN to combat virulence in Pseudomonas aeruginosa
Source: Sci Rep. 2017 Oct 3;7:12615. doi: 10.1038/s41598-017-12667-y (PMC5626770; doi:10.1038/s41598-017-12667-y)
Supplement: Supplementary file 2 — Supplementary Figures [file 41598_2017_12667_MOESM2_ESM.doc]

Targeting the alternative sigma factor RpoN to combat virulence in *Pseudomonas aeruginosa*

Megan G. Lloyd^1^; Benjamin R. Lundgren^2,3^; Clayton W. Hall^4^; Luke B.-P. Gagnon ^4^; Thien-Fah Mah^4^; Jennifer F. Moffat^1^; Christopher T. Nomura^2,3*^

^1^Department of Microbiology and Immunology, SUNY Upstate Medical University, Syracuse, NY, USA

^2^Department of Chemistry, SUNY College of Environmental Science and Forestry, Syracuse, NY, USA

^3^Center for Applied Microbiology, SUNY College of Environmental Science and Forestry, Syracuse, NY, USA

^4^Department of Biochemistry, Microbiology, and Immunology, University of Ottawa, Ottawa, Ontario, Canada

* Corresponding author

E-mail: ctnomura@esf.edu

**
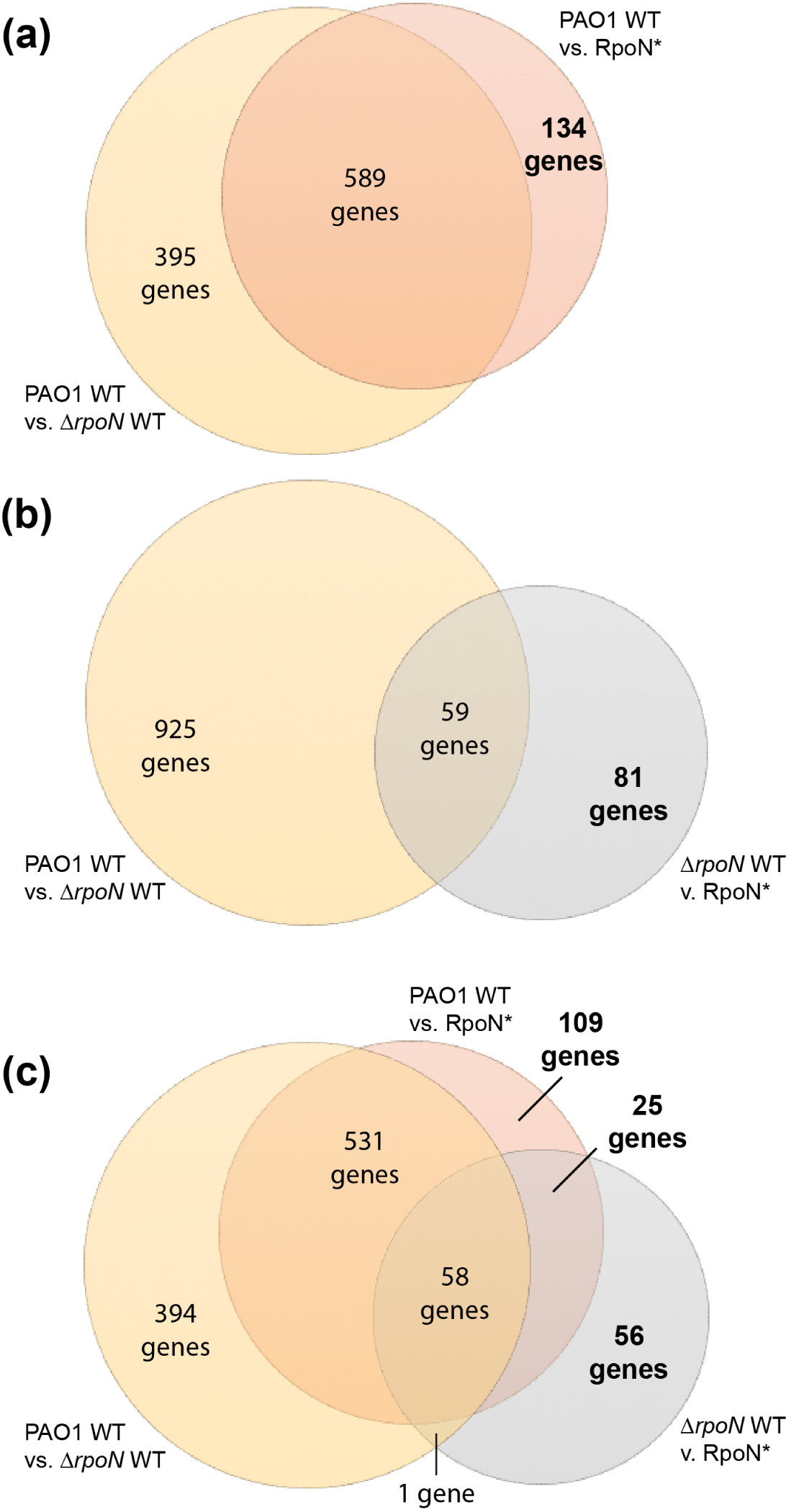
**

**Supplementary Figure S1. Venn diagram comparison of RpoN regulated gene transcription reduced by at least 1.5-fold in *P. aeruginosa*.** (a) Comparison between gene transcription down regulated in *P. aeruginosa* PAO1 and the *P. aeruginosa ΔrpoN* strain each harboring the empty vector plasmid (E.V.). 589 genes overlapped between the two conditions. 134 genes were exclusively regulated by RpoN*. These genes are presumably under some form of dual regulation with other sigma factors. (b) Comparison between gene transcription down regulated in the *P. aeruginosa ΔrpoN* strain either harboring the empty vector plasmid or expressing RpoN*. 59 genes overlapped between the conditions. Transcription of 81 genes was reduced by RpoN* in the *ΔrpoN* strain. RpoN* is presumably reducing transcription by blocking other sigma factors that contribute to dual regulation at these promoters. (c) Comparison of *P. aeruginosa* PAO1 harboring the empty vector plasmid (E.V.), and the *ΔrpoN* strain either harboring the empty vector plasmid or expressing RpoN*. Some genes were down regulated under all conditions, while other genes were exclusive to only one or two conditions. Transcription of 190 genes was reduced only when RpoN* was expressed. These 190 genes are likely regulated by RpoN and at least one other sigma factor.


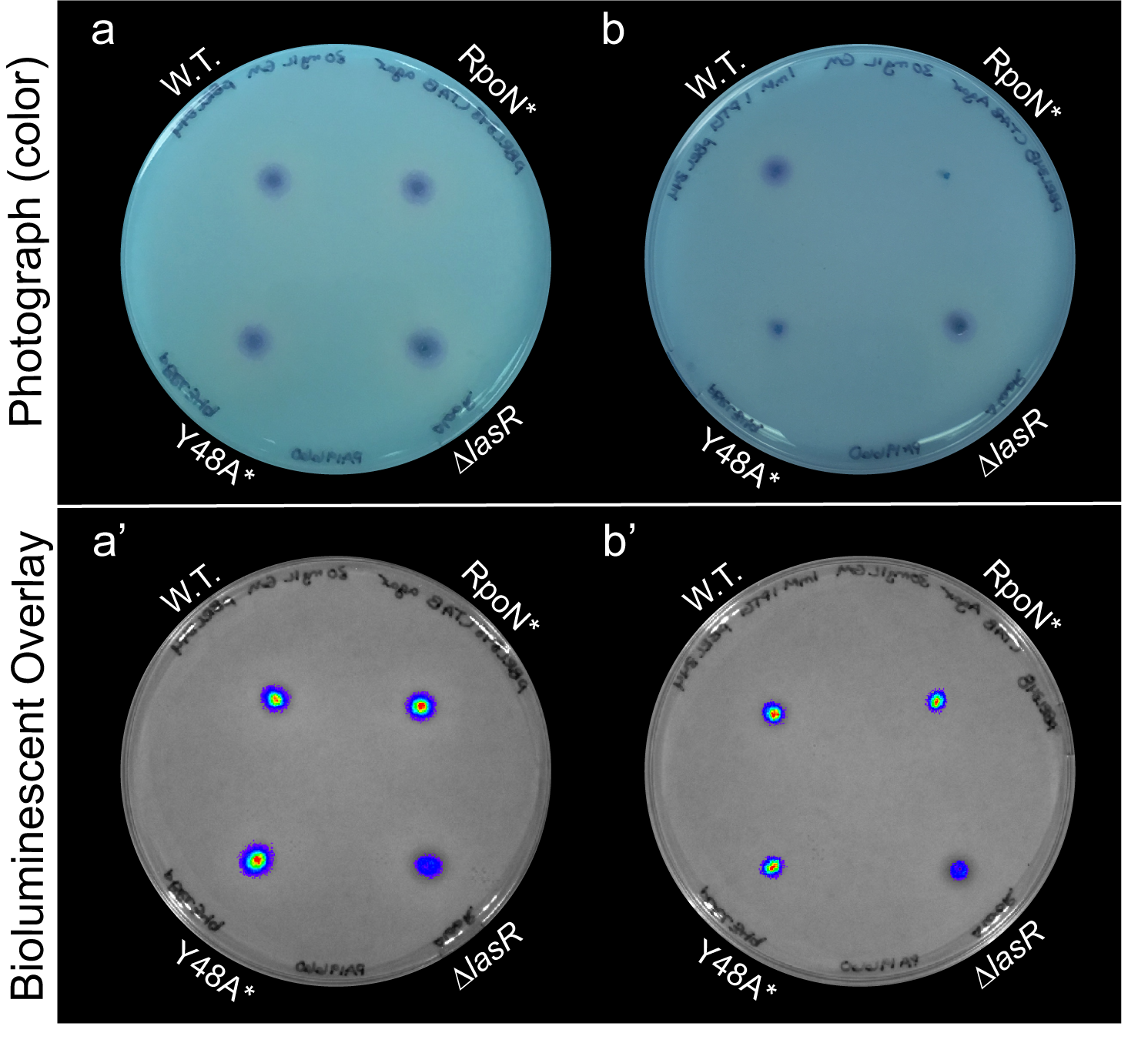


**Supplementary Figure S2. RpoN* decreases rhamnolipid production *in vitro*.** Photograph of rhamnolipid halos on CTAB agar with methylene blue (top) and bioluminescent overlay of the same petri dish showing bacterial growth (bottom). Strains used: *P. aeruginosa* PA19660 wild-type (empty vector), RpoN*, Y48A* point mutant, and *P. aeruginosa* PAO1 Δ*lasR.* All assays were conducted at 37°C for 48 h with 30 mg/L gentamicin, then refrigerated for 48 h prior to imaging. Media either lacked (a, a’) or contained (b, b’) 1 mM IPTG to induce RpoN* and Y48A* expression. Results are representative of the assay performed with n = 10 replicates per condition.


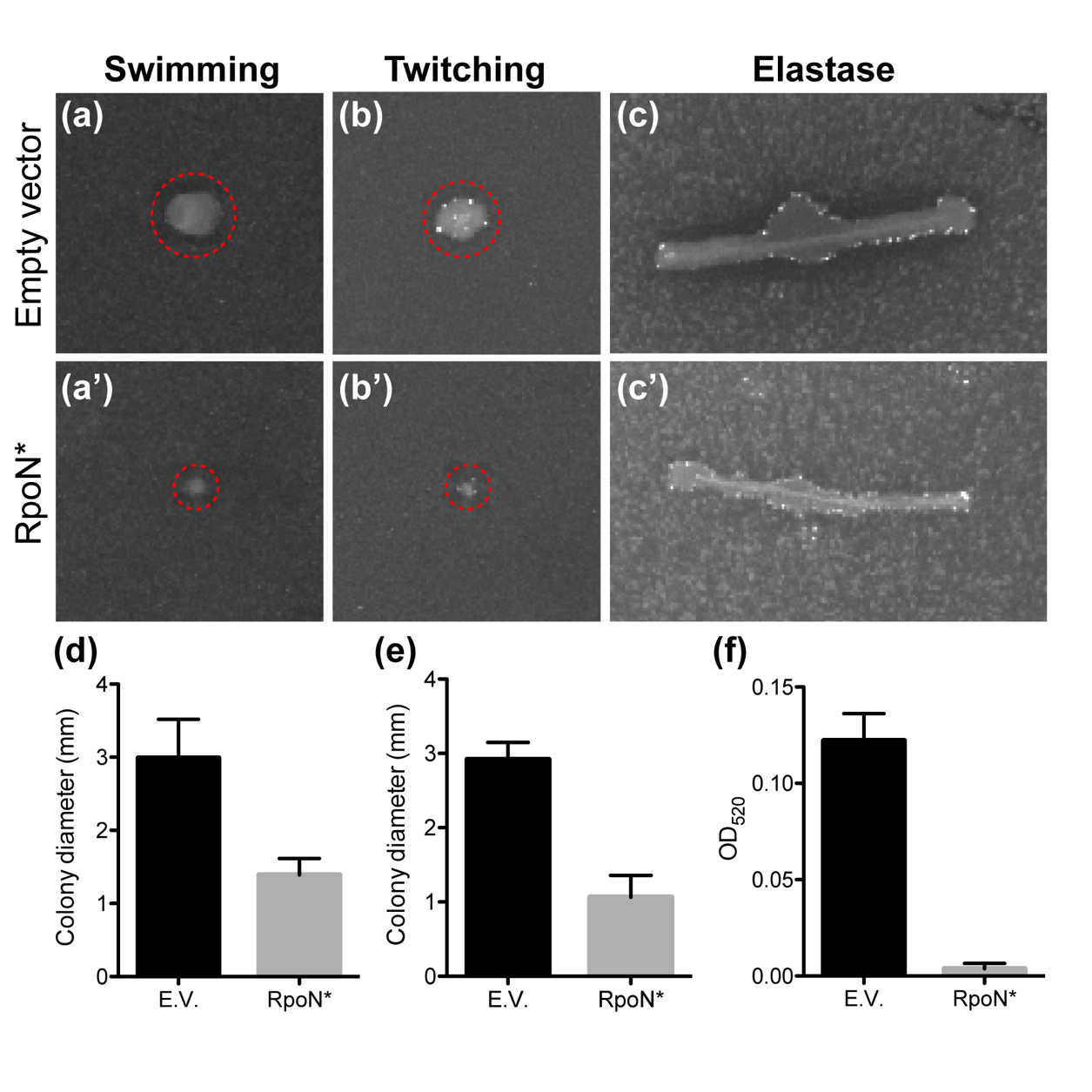


**Supplementary Figure S3. RpoN* reduces *in vitro* virulence-associated phenotypes in *P. aeruginosa* *ΔrpoN*.** (a, a’) Photograph of swimming, or flagellar, motility assay conducted on soft (0.3%) agar. (b, b’) Photograph of twitching, or pili, motility assay conducted on semi-hard (1.3%) agar. (c, c’) Photograph of elastase production conducted on LB agar with elastin agar overlay. Strains used: *P. aeruginosa ΔrpoN* mutant with the empty vector plasmid (a, b, c) and *ΔrpoN* mutant expressing RpoN* (a’, b’, c’). Colony diameter in swimming (d) and twitching (e) motility assays. Assays conducted at 37°C for 24 h in agar containing 30 mg/L gentamicin and 1 mM IPTG. (f) Pyocyanin production assay performed in LB broth containing 30 mg/L gentamicin and 1 mM IPTG. Assay conducted at 37°C, with shaking, for 16 h post-IPTG induction. Data presented as mean ± standard deviation. Students t test performed (*** p ≤ 0.0001). n = 3 to 6 replicates per assay.
