# Supplementary material for: Targeting the alternative sigma factor RpoN to combat virulence in Pseudomonas aeruginosa
Source: Sci Rep. 2017 Oct 3;7:12615. doi: 10.1038/s41598-017-12667-y (PMC5626770; doi:10.1038/s41598-017-12667-y)
Supplement: Supplementary file 3 — Supplementary Note [file 41598_2017_12667_MOESM3_ESM.doc]

Targeting the alternative sigma factor RpoN to combat virulence in *Pseudomonas aeruginosa*

Megan G. Lloyd^1^; Benjamin R. Lundgren^2,3^; Clayton W. Hall^4^; Luke B.-P. Gagnon ^4^; Thien-Fah Mah^4^; Jennifer F. Moffat^1^; Christopher T. Nomura^2,3*^

^1^Department of Microbiology and Immunology, SUNY Upstate Medical University, Syracuse, NY, USA

^2^Department of Chemistry, SUNY College of Environmental Science and Forestry, Syracuse, NY, USA

^3^Center for Applied Microbiology, SUNY College of Environmental Science and Forestry, Syracuse, NY, USA

^4^Department of Biochemistry, Microbiology, and Immunology, University of Ottawa, Ottawa, Ontario, Canada

* Corresponding author

E-mail: ctnomura@esf.edu

**Supplementary Note: Results for Supplementary Fig S3.**

Flagellar structures are lost in *P. aeruginosa ΔrpoN* mutant strains, nearly ablating swimming motility^1,2^. Additionally, pili motility and adhesion are reduced in the absence of native RpoN^2-4^. Here, transcription of genes associated with flagellar structure or function were not affected by the molecular roadblock in the *ΔrpoN* strain. However, transcription of some genes related to twitching or pili-associated motility were reduced. To validate these findings, we evaluated these phenotypes in the *P. aeruginosa ΔrpoN* mutant containing the empty vector or RpoN* plasmid. We expected reduced pili motility but no change in flagellar motility. The *P. aeruginosa ΔrpoN* strain harboring the empty vector had minimal, but measurable, flagellar, or swimming, motility (Supplementary Fig S3a, S3d) and measurable pili, or twitching, motility (Supplementary Fig S3b, S3e). Expression of the molecular roadblock significantly reduced both flagellar and pili motility in the *P. aeruginosa ΔrpoN* strain, which was unexpected (Students t-test, p≤0.0001) (Supplementary Fig 3a’, 3b’, 3d, 3e).

The further reduction in flagellar motility in the *ΔrpoN* strain expressing the molecular roadblock was surprising. However, a possible explanation may be found in other studies. Some genes involved in flagella structure and function have promoter sites for RpoN and FliA or SigX^5^. Furthermore, *fliD*, which encodes a flagellar capping protein, has promoter sites for both RpoN and FleQ, another transcription regulator, and basal flagellar locomotion in the absence of either RpoN or FleQ was possible^6^. Another study indicates a putative FliA binding site at *fliD*^5^. Together, this suggests that in the absence of one regulator, another may partially compensate for the loss. Thus, the molecular roadblock nearly eliminates the basal flagellar motility that occurs in the absence of RpoN by interfering with transcription from other sigma factors and regulatory proteins.

The transcription of pili-associated genes was reduced in the *ΔrpoN* strain and the molecular roadblock further reduced their transcription. These results contradict prior studies that demonstrated a loss of pili and subsequent twitching motility in *P. aeruginosa ΔrpoN* strains^1-4^. Here, twitching motility was observed in the *ΔrpoN* strain, which was significantly reduced by expression of RpoN*. A recent study supports these results, which found many pili-associated genes have multiple sigma factor binding sites in their promoters^5^. Schulz et al. found many pili-associated genes were co-regulated by RpoN and another sigma factor, such as RpoS, RpoH, or FliA, but none by RpoN alone. Furthermore, some pili-associated genes lacked an RpoN binding site^5^. While pili function may be hindered in the *ΔrpoN* strain, it is likely that basal twitching motility is due to dual regulation of many pili-associated genes.

The molecular roadblock also affected gene transcription of the quorum sensing system transcriptional regulator, RhlR. These results were validated by assessing the effects of the molecular roadblock on phenotypes regulated by RhlR. Both the *phz* family, involved in phenazine biosynthesis, and *lasAB*, responsible for elastase production, were downregulated in the microarray analysis. *P. aeruginosa* elastase proteolytically degrades elastin, collagen, and surfactant proteins in the human lung^7-9^. Pyocyanin is a blue-green phenazine pigment and toxin excreted by *P. aeruginosa*. *P. aeruginosa* is resistant to the effects of its own toxin, however other bacteria and eukaryotic cells succumb to its effects^10^. Both elastase and pyocyanin are virulence factors that promote establishment and dissemination of a *P. aeruginosa* infection. Therefore, we conducted an elastase solid media assay and a pyocyanin production assay to evaluate the effects of the molecular roadblock on such virulence factors. To evaluate these virulence-associated phenotypes, the *P. aeruginosa ΔrpoN* mutant containing the empty vector or RpoN* plasmid was used. The *P. aeruginosa ΔrpoN* mutant harboring the empty vector produced elastase, and expression of RpoN* reduced elastase activity (Supplementary Fig S3c and c’). The *ΔrpoN* mutant produced low levels of pyocyanin, which were significantly reduced by expression of RpoN* (Students t-test, p≤0.0001) (Supplementary Fig S3f). Thus, the molecular roadblock reduced the expression of virulence factors in *P. aeruginosa* in the absence of native RpoN.

**References:**

1 Totten, P. A., Lara, J. C. & Lory, S. The rpoN gene product of Pseudomonas aeruginosa is required for expression of diverse genes, including the flagellin gene. *Journal of bacteriology* **172**, 389-396 (1990).

2 Heurlier, K., Denervaud, V., Pessi, G., Reimmann, C. & Haas, D. Negative control of quorum sensing by RpoN (sigma54) in Pseudomonas aeruginosa PAO1. *Journal of bacteriology* **185**, 2227-2235 (2003).

3 Ramphal, R. *et al.* Adhesion of Pseudomonas aeruginosa pilin-deficient mutants to mucin. *Infect Immun* **59**, 1307-1311 (1991).

4 Ishimoto, K. S. & Lory, S. Formation of pilin in Pseudomonas aeruginosa requires the alternative sigma factor (RpoN) of RNA polymerase. *Proc Natl Acad Sci U S A* **86**, 1954-1957 (1989).

5 Schulz, S. *et al.* Elucidation of sigma factor-associated networks in Pseudomonas aeruginosa reveals a modular architecture with limited and function-specific crosstalk. *PLoS Pathog* **11**, e1004744, doi:10.1371/journal.ppat.1004744 (2015).

6 Arora, S. K., Ritchings, B. W., Almira, E. C., Lory, S. & Ramphal, R. The Pseudomonas aeruginosa flagellar cap protein, FliD, is responsible for mucin adhesion. *Infect Immun* **66**, 1000-1007 (1998).

7 Morihara, K., Tsuzuki, H., Oka, T., Inoue, H. & Ebata, M. Pseudomonas Aeruginosa Elastase. Isolation, Crystallization, and Preliminary Characterization. *J Biol Chem* **240**, 3295-3304 (1965).

8 Heck, L. W., Morihara, K., McRae, W. B. & Miller, E. J. Specific cleavage of human type III and IV collagens by Pseudomonas aeruginosa elastase. *Infect Immun* **51**, 115-118 (1986).

9 Mariencheck, W. I., Alcorn, J. F., Palmer, S. M. & Wright, J. R. Pseudomonas aeruginosa elastase degrades surfactant proteins A and D. *Am J Respir Cell Mol Biol* **28**, 528-537, doi:10.1165/rcmb.2002-0141OC (2003).

10 Hassett, D. J., Charniga, L., Bean, K., Ohman, D. E. & Cohen, M. S. Response of Pseudomonas aeruginosa to pyocyanin: mechanisms of resistance, antioxidant defenses, and demonstration of a manganese-cofactored superoxide dismutase. *Infect Immun* **60**, 328-336 (1992).
